# Small molecule metabolites drive plant rhizosphere microbial community assembly patterns

**DOI:** 10.3389/fmicb.2025.1503537

**Published:** 2025-02-11

**Authors:** Yanwei Ma, Heqi Wang, Yalong Kang, Tao Wen

**Affiliations:** ^1^Jiangsu Provincial Key Lab for Organic Solid Waste Utilization, National Engineering Research Center for Organic-based Fertilizers, Jiangsu Collaborative Innovation Center for Solid Organic Waste Resource Utilization, Nanjing Agricultural University, Nanjing, China; ^2^College of Resources and Environmental Science, Yunnan Agricultural University, Kunming, China

**Keywords:** microbiome assembly, microbial community, rhizosphere metabolites, neutral model, ecological process

## Abstract

The assembly of rhizosphere microbial communities is essential for maintaining plant health, yet it is influenced by a wide range of biotic and abiotic factors. The key drivers shaping the composition of these communities, however, remain poorly understood. In this study, we analyzed 108 plant samples and evaluated root traits, plant growth characteristics, soil enzyme activities, rhizosphere metabolites, and soil chemical properties to identify the primary determinants of rhizosphere community assembly. Across 36 soil samples, we obtained 969,634 high-quality sequences, clustering into 6,284 ASVs predominantly classified into Proteobacteria (57.99%), Actinobacteria (30%), and Bacteroidetes (5.13%). Our findings revealed that rhizosphere metabolites accounted for more variance in microbial community composition compared to chemical properties (ANOVA, *F* = 1.53, *p* = 0.04), enzyme activities, or root traits (ANOVA, *F* = 1.04, *p* = 0.001). Seven small molecule metabolites, including glycerol, sorbitol, phytol, and alpha-ketoglutaric acid, were significantly correlated with βNTI, underscoring their role as critical drivers of microbial community assembly. The genus *Rhizobium*, significantly associated with βNTI (R = 0.25, *p* = 0.009), emerged as a keystone taxon shaping community structure. Soil culture experiments further validated that small molecule metabolites can modulate microbial community assembly. The ST treatment, enriched with these metabolites, produced 1,032,205 high-quality sequences and exhibited significant shifts in community composition (Adonis, *p* = 0.001, R = 0.463), with *Rhizobium* showing higher abundance compared to the control (CK). Variable selection (βNTI >2) drove phylogenetic turnover in ST, while stochastic processes (|βNTI| < 2) dominated in CK. This study provides quantitative insights into the role of rhizosphere metabolites in shaping microbial community assembly and highlights their potential for targeted modulation of rhizosphere microbiomes.

## 1 Introduction

Rhizosphere microbiome plays a crucial role in promoting plant health and growth by offering protection against pests and diseases, enhancing nutrient uptake, and helping plants in coping with environmental stresses (Berendsen et al., 2012; Pérez-Jaramillo et al., 2016; Wen et al., 2022; Yue et al., 2023; Xun et al., 2024). In recent years, a multitude of researchers across various fields have endeavored to comprehend the fundamental mechanisms that govern the composition, dynamics, and assembly of the rhizosphere microbiome (Edwards et al., 2015; Zhong et al., 2019; Chen et al., 2020; Kang et al., 2021a; Adedayo et al., 2022; Wen et al., 2022; Malacrinò and Bennett, 2024; Wang S. et al., 2024). Assembly of the rhizosphere microbiome is influenced by a multitude of factors, encompassing abiotic factors such as rhizosphere metabolites (Zhalnina et al., 2018; Chen et al., 2020; Wen et al., 2020), soil types and their physicochemical properties (Edwards et al., 2015; Zhang et al., 2019; Wu et al., 2023), along with climate variables (Aslam et al., 2022), as well as biotic factors including disease and insect pests (Benitez et al., 2017; Berendsen et al., 2018), plant species and genotypes (Edwards et al., 2015; Oyserman et al., 2022), root phenotypes (Cotton et al., 2019; Rüger et al., 2021), plant developmental stages and statuses (Zhang et al., 2018; Adedayo et al., 2022), and the diversity and composition of resident microbiota (Malacrinò and Bennett, 2024). However, there remain significant challenges in quantitatively assessing how various factors influence the assembly of the rhizosphere microbial community.

Rhizosphere metabolites play a crucial role in shaping the assembly of rhizosphere microbiomes and facilitating the plant-microbe interaction (Bi et al., 2022; Jiang et al., 2024), as they are subject to modification in quantity and composition by the above-mentioned abiotic and biotic factors. The metabolites in the rhizosphere are predominantly derived from the secretions of plant roots and the activities of soil microorganisms (Jain et al., 2024; Jiang et al., 2024; Liu et al., 2024). Specific rhizosphere metabolites have been recognized as chemical signals that attract specific microbial taxa from the bulk soil to the rhizosphere (Zhou et al., 2023). For instance, the presence of metabolites (e.g., ribose, lactic acid, xylose, mannose, maltose, gluconolactone, and ribitol) in tomato root exudate serves to attract antifungal soil commensal bacteria, thereby facilitating the inhibition of pathogen invasion (Wen et al., 2022). The interactions between rhizosphere metabolites and the microbiome plays a vital role in influencing multiple facets of plant health and growth, and even productivity, such as the control of soil-borne diseases and the alteration of root growth (Wen et al., 2023; Zhou et al., 2023; Jiang et al., 2024; Liu et al., 2024). Therefore, comprehending the dynamics and functions of rhizosphere metabolites is essential for uncovering the microbial-driven mechanisms that contribute to the advantages of agricultural land management practices.

Fertilization is recognized as a fundamental management practice with significant effects on soil fertility, crop development, root system architecture, rhizosphere microbial communities and assembly, and yield in agricultural production (Shen et al., 2019; Kang et al., 2021a; Deng et al., 2022; Kang et al., 2022; Liu et al., 2024). Chemical fertilizers (synthetic chemical or mineral raw materials) and bio-organic fertilizers (mixed organic substances and beneficial microorganisms) are the most valued in daily agricultural production activities (Kang et al., 2021b; Wang et al., 2022; Li et al., 2023). Recently, some studies have suggested that (bio-)organic fertilizers promote deterministic processes in the construction of microbial communities (Dong et al., 2021; Chen et al., 2023), whereas chemical fertilizers subject the community construction of microbial taxa to stochastic processes (Dong et al., 2021; Hou et al., 2022). However, there is considerable debate surrounding the impact of fertilization (both organic and inorganic) on the relative significance of stochastic and deterministic processes (Wang et al., 2017; Shi et al., 2020; Zhu et al., 2024). For instance, Zhu et al. (2024) suggested that stochastic processes were the primary determinant of bacterial community assembly in *Suaeda salsa* salt marsh under the application of organic fertilizer. Indeed, aside from fertilization factors, numerous other variables such as soil properties (e.g., pH, salt and carbon-N ratio) (Wang et al., 2017; Wu et al., 2023; Zhu et al., 2024), rhizosphere metabolites (Wu et al., 2017), and the extent of microbial diversity (Xun et al., 2019) also play a significant role in influencing these two processes. Hence, a comprehensive examination of the assembly of microbial communities and the factors that influence them following various fertilizer applications is essential for advancing our understanding of the mechanisms underlying of plant-microbe-soil interactions.

Cucumber is one of the prominent economical vegetable crops cultivated in greenhouses worldwide. According to reports, China’s cucumber planting area represents approximately 56% of the global total, with its production contributing to over 80% of the worldwide cucumber production (Feng et al., 2020). However, the excessive application of chemical fertilizers has led to various planting challenges, including soil acidification, nutrient imbalances, reductions in soil microbial diversity, and exacerbation of soil-borne pathogens, ultimately hindering the sustainable development of greenhouse vegetable production (Qiu et al., 2012; Zhang et al., 2013; Cai et al., 2017; Wang et al., 2023). In order to mitigate these challenges, bio-organic fertilizers have been employed to modify the composition of rhizosphere microbiota and/or metabolites with the aim of inhibiting soil-borne pathogen populations, boosting cucumber plant vitality, and ultimately enhancing cucumber quality and yield (Zhang et al., 2013, Cai et al., 2017, Wang et al., 2023). However, the mechanisms underlying the rhizosphere microbiome-metabolites interactions, as well as the key factors driving the assembly of the rhizosphere microbiome of cucumber in response to different fertilizer managements, have not been fully elucidated.

To this end, we conducted a one-year pot experiment at the Baima Test Base of Nanjing Agricultural University in Jiangsu province, China under no fertilizer (NF, as control), chemical fertilizer (CF), and bioorganic fertilizer (BIO) treatments to measure factors including plant growth properties, root morphological characteristics, edaphic factors and enzyme activities, and rhizosphere metabolites composition. We aimed to address (1) whether rhizosphere metabolites are the key drives in the rhizosphere community assembly processes and which types of exudate compounds are responsible; (2) whether specific microbial community responded to the those factors; and (3) whether rhizosphere metabolites could be used as regulators for targeted modulation of rhizosphere microbial community assembly processes.

## 2 Materials and methods

### 2.1 Study site, substrates, and materials

**Figure 1:**
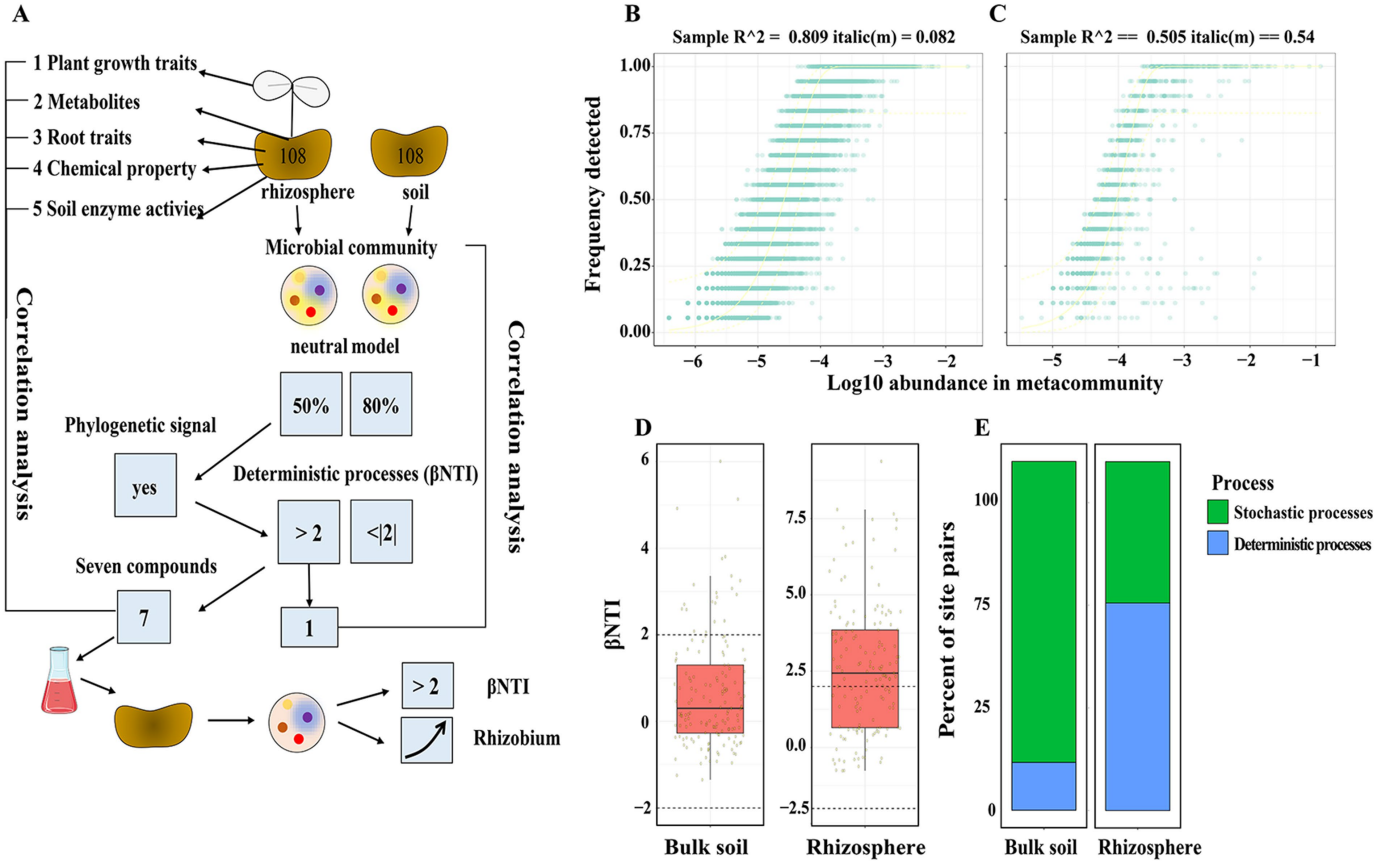
Contributions of deterministic and stochastic processes on rhizosphere community assembly. βNTI measures phylogenetic turnover. **(A)** Diagram of the experiment workflow. **(B,C)** The probability of detecting an ASVs in any given sample is related to its relative abundance. Solid line indicates the path of a fitted model. Dashed lines indicate the boundaries of the 95% prediction interval. **(D)** Contributions of deterministic and stochastic processes on community assembly within pot experiment. Dashed lines indicate the significance thresholds for βNTI. **(E)** The relative influence of each community assembly process of bulk and rhizosphere soil samples from pot experiment.

In October 2019, approximately 200 kg of soil (0 to 20 cm deep), was collected from Jianning County, Sanming city, China (34°04’ N, 108° 10′ E) with no history of cucumber cultivation. The red soil type is classified as Ferrosols-Udic in the Chinese Soil Taxonomy (Huang et al., 2017). The soil samples were air-dried in a natural manner, sieved through a 5 mm mesh to eliminate plant debris and rocks, homogenized, and subsequently stored in plastic bags at room temperature prior to utilization. From 20th March 2020 to 20th May 2020, we carried out a pot experiment (Figure 1A) at the Nanjing Agricultural University Test Base (31°36’ N, 119°10′ E), Baima Town, Nanjing, Jiangsu Province. The climate type at test site is the transition zone from north subtropical to mid-subtropical. The climate is mild and humid, the annual average for temperature is 15.4°C, rainfall is 1087.4 mm, and the frost-free period is 237 d. Before the experiment, the soil properties were: the pH value is 4.65, soil organic matter content (SOM) is 18.2 g kg^−1^, total nitrogen content (TN) is 0.96 g kg^−1^, available potassium content (AK) is 122.7 mg kg^−1^ and available phosphorus content (AP) is 74.3 mg kg^−1^.

The seeds of cucumber cultivar (*Cucumis sativus* L.), “Feng-Lu,” a commercial variety in China, were purchased from Jiangsu Academy of Agricultural Sciences. On March 20, 2020, the cucumber seeds were surface sterilized with 75% ethanol for 30 s followed by 5% NaClO for 5 min before planting. The sterilized seeds were placed in Petri dishes with wet autoclaved filter paper in a growth chamber (25°C, 70% relative humidity in the dark). After 2 days of pregermination, the cucumber seedlings (two true-leaf stage) were transplanted to plastic pots (15 cm diameter, 20 cm height) with 3 kg of soil.

### 2.2 Greenhouse pot experiments

The three treatments were as follows: (1) CK, no fertilizer control; (2) CF, chemical fertilizer treatment; and (3) BIO, bioorganic fertilizer treatment. CF treatment was consisting of analytically pure urea (N 46%), calcium superphosphate (P_2_O_5_ 15%), and potassium sulphate (K_2_O 50%). Bioorganic fertilizer (pH 6.6, N 4.77%, P_2_O_5_ 2.26%, K_2_O 1.00%, organic matter content 40%) was purchased from Jiangsu Lianye Fertilizer Co., Ltd., (the colony count of *Bacillus amyloliquefaciens* SQR9 was 1 × 10^8^ CFU g^−1^ dry weight). Based on the equal nutrient content and nutrient requirements of cucumber (N: P_2_O_5:_ K_2_O = 1:0.5:1), CF and BIO treatments were adjusted to the same amounts of N, P and K according to the nutrients of BIO treatment if necessary by urea, calcium superphosphate, and potassium sulphate, respectively. The amount of nitrogen, phosphorus and potassium in each fertilized pot was 2.86 g N, 1.36 g P_2_O_5_ and 2.86 g K_2_O, respectively. Thus, each BIO chamber received 60 g (2% w/w) bioorganic fertilizer and 2.26 g potassium sulphate. Each CF chamber was supplied 6.22 g urea, 9.07 g calcium superphosphate, and 5.72 g potassium sulphate. Bioorganic fertilizer and calcium superphosphate were used as basal fertilizers on November 25, 2019. The urea and potassium sulphate was applied on March 28, 2020. There were 36 seedlings of each treatment, for a total of 108 pots (Figure 1A); the pots were then randomly placed in a growth chamber (28/26°C day/night cycle, 70% relative humidity, and 180 μmol light m^−2^ s^−1^) and irrigated as needed. On 20th May 2020, 6 pots were randomly pooled into one sample, and thus 6 replicates from each treatment. In total, 18 samples were collected. The comprehensive workflow of our research is depicted in Figure 1A.

### 2.3 Plant growth traits measurement

On 20th May 2020, plant growth traits including height (cm), stem thickness (D, cm), relative growth rate (RGR), leaf area (LA), and root traits [e.g., average lateral root diameter (LRAD), total lateral root number (TLRN), total lateral root length (TLRL), mean inter-branch density (MID), maximum order of lateral roots (MaxO), and branching density (BI, ration of branches number to root length)] were measured following previous protocols (Kang et al., 2021b). The methods used for collecting the plant samples are detailed in the Supplementary Method S1.

### 2.4 Rhizosphere soil sampling and chemical properties analysis

Rhizosphere soil was collected and subjected to the collection of root samples on May 20, 2020. In summary, the loosely attached soil on the cucumber roots was removed and discarded, while the soil that remained tightly adhered was collected as rhizosphere soil (Wen et al., 2020). Concurrently, bulk soil samples were collected. A portion of approximately 180.0 g of soil was air-dried for the determination of soil physicochemical properties, whereas another portion of approximately 10.0 g was stored at 4°C for the analysis of soil extracellular enzyme activities. The remained part of about 10.0 g was stored at −80°C and used for the microbial analyses. Detailed procedures for determining those properties are described in Supplementary Method S2.

### 2.5 DNA extraction, sequencing, and data processing

About 0.5 g rhizosphere soil was used to extract the Genomic DNA by using the PowerLyzer PowerSoil DNA Isolation Kit (Qiagen, Germany) in accordance with the manufacturer’s instructions. Bacterial V4 region for amplicon sequencing, and the primers 515F: GTGYCAGCMGCCGCGGTAA and 806R: (GGACTACNVGGGTWTCTAAT) (Walters et al., 2015) to yield an amplicon of 292 bp. Detailed procedures for PCR amplification are described in Supplementary Method S3.

### 2.6 Rhizosphere metabolome detection by GC–MS

In this study, the “rhizosphere metabolome” refers to the collective pool of small molecule metabolites in the rhizosphere, including those released by cucumber roots and produced by the microbial community. Soil samples were divided into two portions (0.2 g each) and transferred into 2 mL EP tubes. Subsequently, 24 μL of Adonitol solution (1 mg mL^−1^ in dH₂O) was added as an internal standard for rhizosphere metabolome extraction. The detailed extraction procedures are provided in Supplementary Method S4. The GC-TOF-MS (gas chromatography-time of flight mass spectrometry) analysis and the analysis of the raw peak performed as reported by Wen et al. (2020).

### 2.7 Impacts of seven small molecule metabolites on the rhizosphere microbiome assembly process

In a soil application trial, seven low molecular weight metabolites (ST; glycerol, Sorbitol, Phytol, 1,2,4-Benzenetriol, succinate semialdehyde, alpha-ketoglutaric acid and D-Glyceric acid) were tested for their effects on the assembly of soil microbial communities. Detailed procedures for incubating are described in Supplementary Method S5. Finally, 12 samples for the two treatments (2 treatments × 6 soil samples) were obtained and stored at −80°C.

The primers 341F/806R (F: CCTAYGGGRBGCASCAG; R: GGACTACHVGGGTWTCTAAT) were used to amplify the V3–V4 region of the 16S rRNA gene of the bacterial communities from the 12 collected samples with an amplicon size of 465 bp. The target sequences were identified from the raw sequences by aligning them with the 515F/806R primers, ensuring that they corresponded to the same region of the 16S rRNA gene as observed in the rhizosphere samples (Wen et al., 2020). Afterward, the PCR amplification and sequencing of the 16S rRNA were conducted in a manner consistent with the procedures outlined previously.

### 2.8 Statistical analyses

The alpha diversity of microbial communities was assessed through statistical analysis and visualization, utilizing metrics such as Richness, Shannon, and Pielou evenness. This analysis was conducted on the Amplicon Sequence Variant (ASV) table with the minimum read number across all samples, and the results were visually represented using boxplots. Next, the relative abundance of each ASV was standardized using the normalize_table.py script in Qiime (version 1.9.1) (Caporaso et al., 2010), followed by the preparation of Bray–Curtis similarity matrices using the beta_diversity.py script. Principal coordinate analysis (PCoA) plots were produced by utilizing Bray–Curtis similarity matrices generated with the R package ggplot2 (Wen et al., 2020). For beta diversity, PERMANOVA (Adonis, transformed data by Bray-Curtis, permutation = 999) (Gower, 1966) was used to determine the similarity of bacterial communities among treatments. Network analysis utilizing the Spearman correction and its associated properties were computed using the “igraph” package (Wen et al., 2022). All analyses and graphical visualizations were performed in R Environment using libraries dplyr, reshape2 and ggplot2. A nonparametric *t*-test was used to determine significant differences in alpha diversity, metabolites and soil properties among treatments using the “EasyStat” package in R with a false discovery rate (FDR).

Here, we used two approaches to examine bacterial community assembly. First, we applied the neutral model described by Merifield and Sloan (2006). Second, we estimated the influence of deterministic processes on community assembly by calculating the *β*-nearest taxon index (βNTI) between pairs of samples as described in Stegen et al. (2012). Detailed procedures for analyzing the bacterial community assembly are described in Supplementary Method S6.

In order to investigate the impact of dispersal on community assembly processes, we assessed ASV turnover using the abundance-weighted Raup-Crick metric (RCbray), following the methodology outlined by Stegen et al. (2013). RCbray assesses whether ASV turnover among sites differs from what would be expected based on ecological drift alone. Initially, the Bray–Curtis dissimilarity was calculated for each sample pair. Subsequently, a null-model community with equivalent size and richness was randomly generated for each sample. Every null model is created by randomly selecting ASVs (weighted by frequency across all samples) and assigning their relative abundance based on their relative abundance in the metacommunity. The Bray–Curtis dissimilarity between all pairs of simulated communities is subsequently calculated. This procedure is iterated 999 times to produce a null model distribution. The Relative Contribution of Bray–Curtis dissimilarity (RCbray) was determined through a calculation involving the summation of the number of simulated communities exhibiting a Bray–Curtis dissimilarity greater than the observed dissimilarity (Nsim>obs), half of the number of simulated communities with a Bray–Curtis dissimilarity equal to the observed dissimilarity (Nsim = obs), and subsequent division by the total number of simulations (999). Sample pairs with a |βNTI| < 2 and an |RCbray| > 0.95 suggest that bacterial community turnover is primarily driven by stochastic processes. Conversely, the bacterial community turnover is primarily driven by deterministic processes (Stegen et al., 2012). To determine the relative proportions of community assembly governed by deterministic and stochastic processes within each sample, we combined the results of the βNTI and RCbray analyses.

Additionally, the raw sequence data reported in this paper have been deposited in the Genome Sequence Archive of the BIG Data Center, Chinese Academy of Sciences, under accession code PRJCA034325.

## 3 Results

### 3.1 Phylogenetic turnover in the rhizosphere is largely deterministic and in soil is random

A total of 969,634 high-quality sequences were obtained across 36 soil samples, including both bulk soil and rhizosphere samples. The average read count for each sample was 35,824 (standard deviation (SD) 2,388). All sequences were clustered into 6,284 ASVs. The majority of ASV belonged to the phyla *Proteobacteria* (57.99% with sd 0.065), *Actinobacteria* (30% with sd 0.051), *Bacteroidetes* (5.13% with sd 0.02), *Firmicutes* (2.01% with sd 0.014), Unassigned (1.71% with sd 0.005), *Chloroflexi* (1.68% with sd 0.008), *Acidobacteria* (0.51% with sd 0.003) *Verrucomicrobia* (0.32% with sd 0.002), *Planctomycetes* (0.28% with sd 0.002) and *Spirochaetes* (0.13% with sd 0.001).

The following section will examine two approaches to the study of bacterial community assembly. The initial step was to assess the extent to which stochastic processes are involved in the assembly of soil and rhizosphere bacterial communities. The neutral model, which accounts for 80.9% of the variation in ASV detection frequency, was estimated to have an estimated migration factor (m) of 0.26 in bulk soil (Figure 1B). In rhizosphere soil, the neutral model accounted for 50.5% of the variation in ASV detection frequency, with an estimated migration factor (m) of 0.54 (Figure 1C). The first step in the study was to assess the phylogenetic signal in habitat preference based on root traits, plant growth traits, rhizosphere metabolites, and soil chemical properties. A notable positive correlation was found between phylogenetic distance and habitat preference in closely related taxa (Supplementary Tables S2, S3), thus validating the assumptions of the βNTI metric. What’s more, deterministic processes (βNTI >2) was observed in the rhizosphere, while stochastic process (|βNTI| < 2) dominate phylogenetic turnover in bulk soil (Figures 1D,E).

### 3.2 Rhizosphere metabolites were more associated with the rhizosphere microbiome rather than other factors

**Figure 2:**
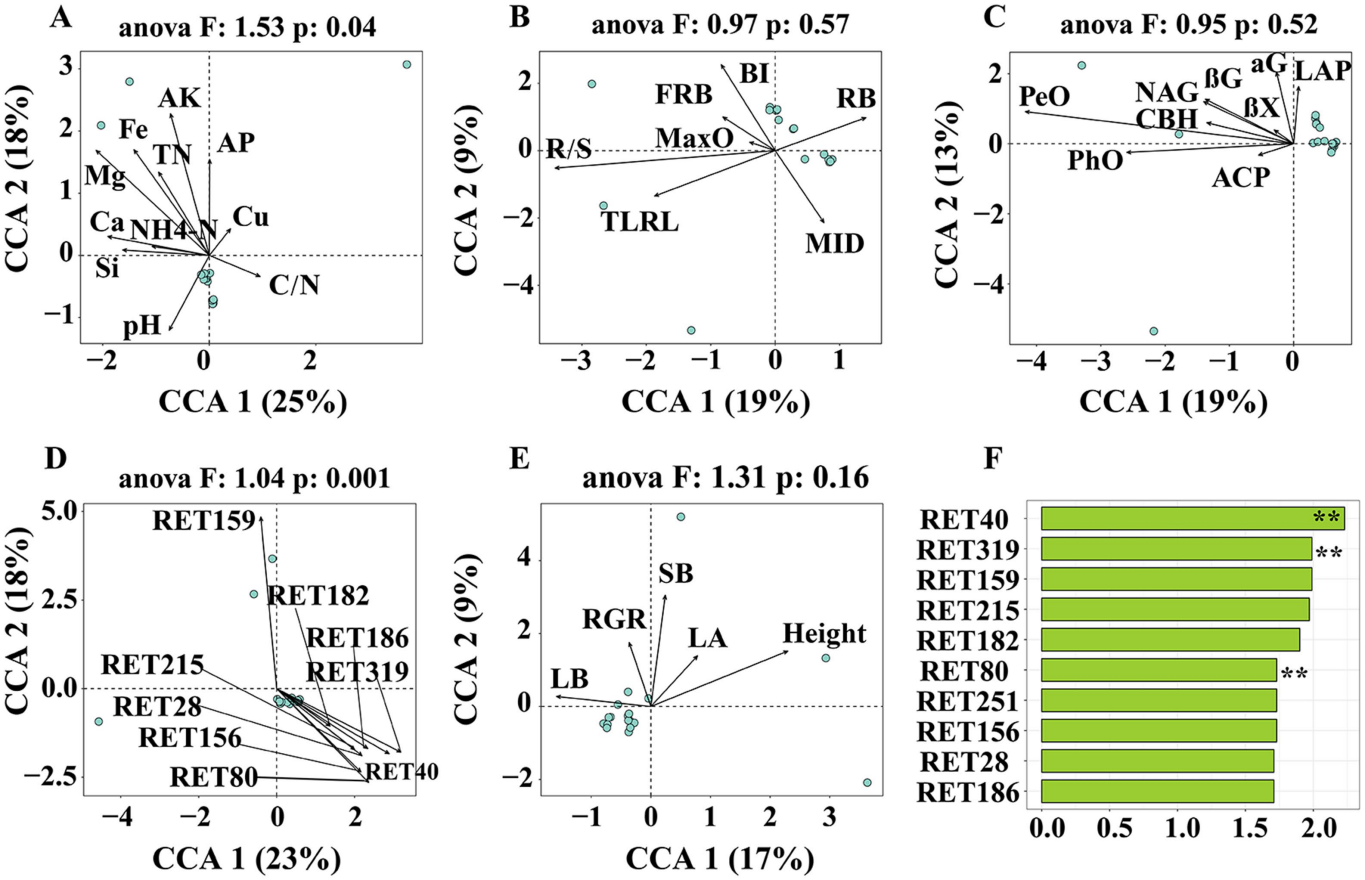
Ordinations were RDA/CCA analysis based on Bray-Curtis distance representing the effect of **(A)** chemical properties, **(B)** root traits, **(C)** soil enzyme activities, **(D)** rhizosphere metabolites, and **(E)** plant growth traits to rhizosphere microbiome. **(F)** Ordination of variable importance derived from the random forest (RF) models for the rhizosphere metabolites. SOC, soil organic carbon; TN, total nitrogen; AP, available phosphorus; AK, available potassium; NH4-N, ammonium nitrogen; Ga, exchangeable sodium concentration; Mg, exchangeable magnesium concentration; Si, available silicon; Fe, available iron; Cu, available copper; C/N, ration of SOC to TN. TLRN, total lateral root length, MID, mean inter-branch density; MaxO, maximum order of lateral roots; BI, branching density; FRB, fine root biomass; RB, root biomass; R/S, ration of root biomass to shoot biomass. αG, α-1,4-Glucosidase; βG, β-1,4-Glucosidase; βX, β-1,4-xylosidase; CBH, β-D-Cellobiohydrolase; LAP, leucine amino peptidase; NAG, β-1,4-N-Acetyl-glucosaminidase; ACP, acid phosphomonoesterase; PeO, Peroxidas; PhO, Phenol oxidase. LB, leaf biomass; SB, shoot biomass; LA, leaf area; RGR, relative growth rate.

Examination of the rhizosphere metabolome using GC-TOF-MS resulted in the detection of 323 chromatographic peaks, with 172 compounds identified in all samples. These included 14 amino acids and amides, 7 amines, 2 Pyridines, 26 alcohols, 61 acids, 4 phenols, 1 Alloxazine, 1 Purine, 4 aldehyde, 1 Alkaloids, 27 sugars, 34 sugar acids, 11 ketones, 1 Adenosines, 8 esters and 3 others (Supplementary Figure S1). Moreover, RDA/CCA showed that soil chemical properties (ANOVA, *F* = 1.53, *p* = 0.04) and important rhizosphere metabolites (ANOVA, *F* = 1.04, *p* = 0.001) were significantly affected bacterial composition at the ASVs level, while enzyme activities, root traits and indicators of aboveground growth were not significantly (Figures 2A–E). Mantel tests showed root exudates were a significant correlation (R = 0.397, *p* = 0.001) with rhizosphere microbiome. In contrast, no correlation was found between chemical properties, enzyme activities and root traits with rhizosphere microbiome (Supplementary Figure S2). Based on the random forest model, intriguingly, the rhizosphere microbiome was significantly correlated with glycerol (RET40), 7,8-Dimethylalloxazine (RET 319) and Capric Acid (RET80) (Figure 2F).

### 3.3 Small molecule metabolites explain the rhizosphere assembly process mediated by the genus *Rhizobium*

**Figure 3:**
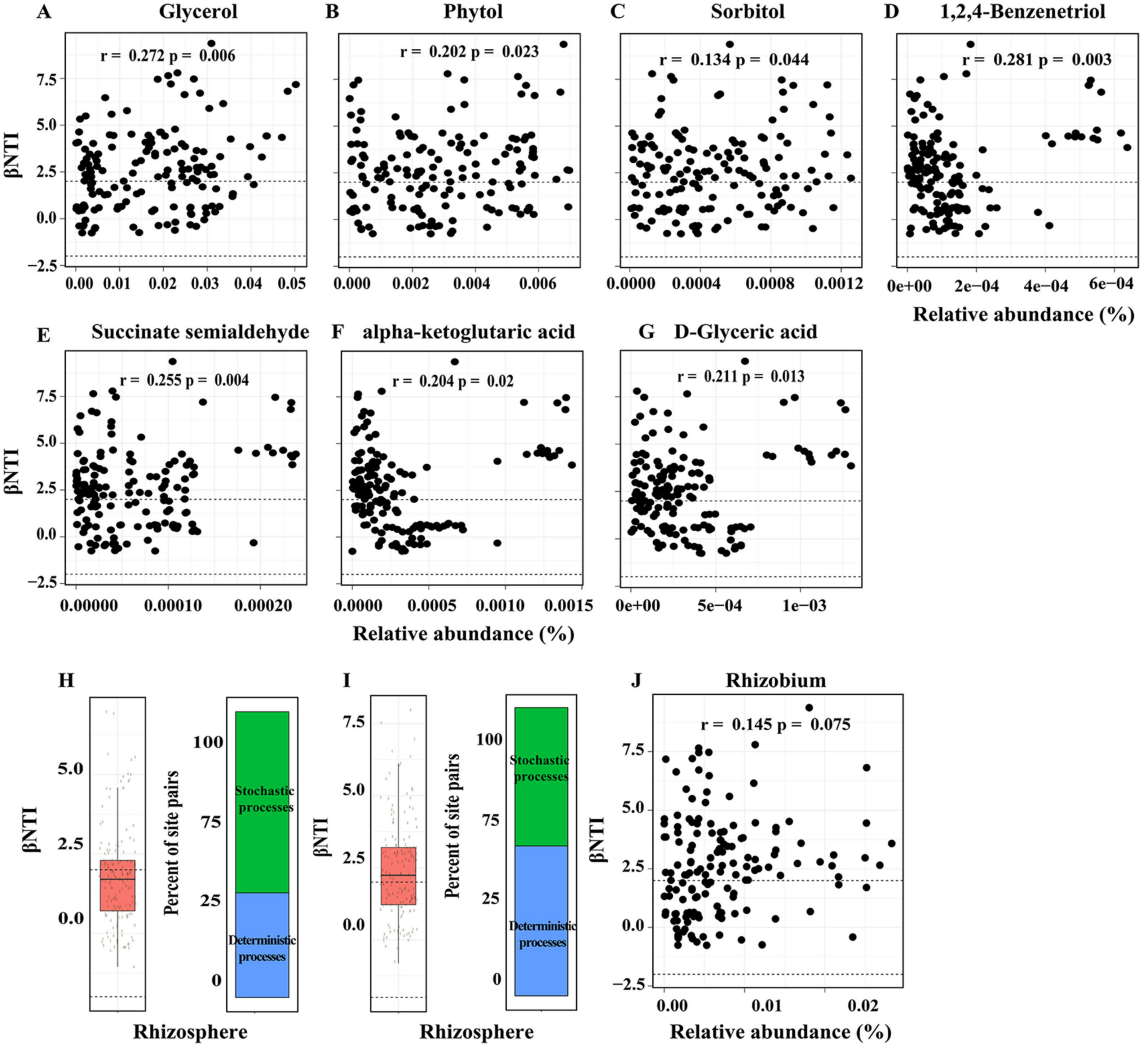
**(A-G)** Differences in rhizosphere metabolites explain significant variation in phylogenetic turnover. βNTI and RCbray were calculated by the microbial community, which removed Genus of Rhizobium **(H)** and anyone Genus randomly **(I)**. βNTI < -2 indicates a significant effect of homogeneous selection, βNTI > 2 indicates a significant effect of variable selection. and |βNTI| < 2 indicates a lack of selection and, therefore, dominance of stochastic processes. Dashed lines indicate the significance thresholds for βNTI. **(J)** Mantel test were used to found the correction between βNTI and Genus of Rhizobium.

In order to ascertain the extent to which these properties account for community assembly patterns, we conducted analyses to assess the correlations between βNTI and various chemical properties, enzyme activities, root traits, and rhizosphere metabolites. There was no significant correlation between each index of chemical properties, enzyme activities and root traits with βNTI (Supplementary Figure S3). Subsequently, a correlation analysis was conducted between 11 groups of rhizosphere metabolites and βNTI, which identified a significant correlation between Phytol and βNTI (R = 0.25, *p* = 0.009) (Supplementary Figure S4). Furthermore, 26 compounds belonging to Phytol were analyzed for correlation with βNTI, revealing that only three compounds—glycerol (RET40), sorbitol (RET46), and Phytol (RET44)—showed significant correlations (Supplementary Figure S5). In order to ensure comprehensive coverage of potentially influential compounds in the microbiome assembly process, compounds exhibiting a coefficient of variation (CV) greater than 0.5 were selected for correlation analysis with βNTI. The results indicated that seven compounds (1,2,4-Benzenetriol, succinate semialdehyde, alpha-ketoglutaric acid, D-Glyceric acid, Glucoheptonic acid, 2-amino-2-methylpropane-1,3-diol, and 2-Butyne-1,4-diol) displayed significant correlations with βNTI (Figure 3A; Supplementary Figure S6).

In the investigation of core taxa during microbial community assembly, genera with a relative abundance greater than 0.01 were selected for correlation analysis with βNTI. The results indicated a significant correlation between the genus *Rhizobium* and βNTI (Supplementary Figure S7), prompting the removal of false positive correlations between taxa with high non-zero values and βNTI. Subsequent recalculation of βNTI and RCbray after excluding the genus *Rhizobium* revealed that stochastic processes (|βNTI| < 2) predominantly govern phylogenetic turnover (Figure 3H). Finally, in order to enhance the validity of the findings, βNTI and RCbray were recalculated iteratively by systematically excluding each Genus (except *Rhizobium*) individually, with a focus on instances where the mean value of βNTI >2 (Figures 3I,J).

### 3.4 Seven small molecule metabolites are regulators for targeted modulation of soil community assembly processes

**Figure 4:**
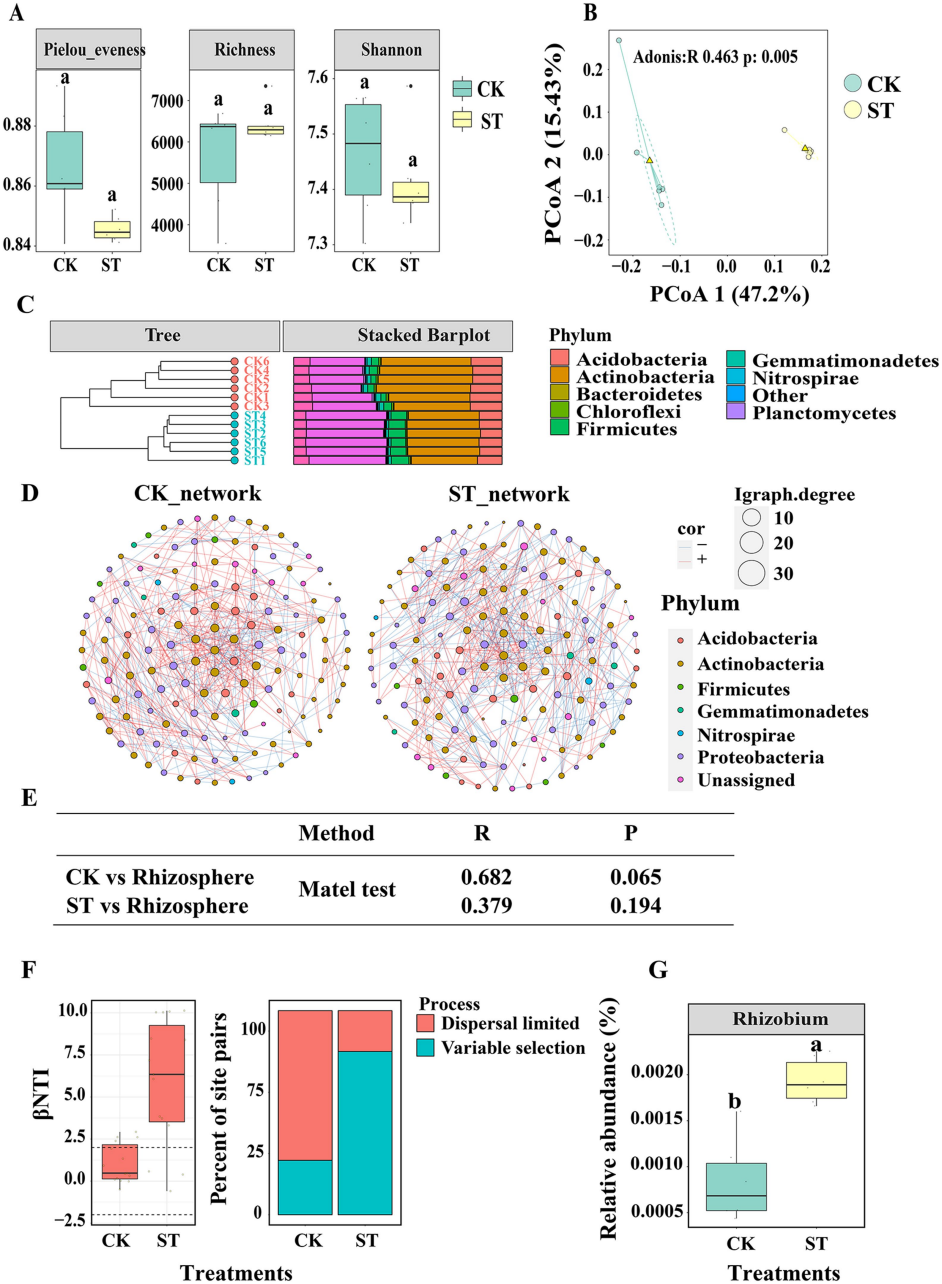
**(A)** Illustrates the alpha diversity of soil bacterial communities, respectively. The horizontal bars within boxes represent the median. The tops and bottoms of boxes represent 75th and 25th quartiles, respectively. All outliers were plotted as individual points. **(B)** The principal coordinates analysis (PCoA) with Bray-Curtis dissimilarity performed on the taxonomic profile (at the ASVl evel) bacterial communities. **(C)** The relative abundance (%) of the major phyla present in the bacterial community and the dendrogram groups samples by hierarchical clustering based on microbial community similarity. **(D)** the co-occurrence networks of the abundance ASV (ASVs with the top abundance 150). Edges represent significant Spearman correlations (ρ > |0.8|, P < 0.05). Light blue lines represent a significant negative correlation, and light red lines represent a significant positive correlation. **(E)** The similar of microbial communities between BT/CK and rhizosphere calculated by mantel test. **(F)** βNTI and RCbray were calculated by microbial community of CK and ST. **(G)** The relative abundance of Rhizobium in CK and ST. Different lowercase letters signify significant differences between CK and ST based on the LSD test (*p*  < 0.05).

Seven small molecule metabolites, including glycerol, sorbitol, phytol, 1,2,4-benzenetriol, succinate semialdehyde, alpha-ketoglutaric acid, and D-glyceric acid, were ultimately chosen for the soil culture experiment based on a selection criterion of R > 0.2. We successfully acquired 1,032,205 high-quality sequences from a combined 12 soil samples, encompassing both control (CK) and treatment (ST) groups. The average read count for each sample was determined to be 86,017 (SD 20476). All sequences were clustered into 15,126 ASVs. The majority of ASV belonged to the phyla *Actinobacteria* (38.12% with sd 0.05) *Proteobacteria* (32.33% with sd 0.06), *Acidobacteria* (12.92% with sd 0.021), *Firmicutes* (5.49% with sd 0.024) Unassigned (3.62% with sd 0.038) *Gemmatimonadetes* (1.56% with sd 0.005), *Chloroflexi* (0.75% with sd 0.002), *Nitrospirae* (0.47% with sd 0.001), *Planctomycetes* (0.36% with sd 0.003) and *Bacteroidetes* (0.3% with sd 0.001). However, there were no significant differences in *α*-diversity, as indicated by Chao1, Shannon, and Pielou evenness values, between the CK and ST (Figure 4A). A higher prevalence of *Planctomycetes* was observed in the ST treatment, whereas *Actinobacteria* were more abundant in the CK treatment (Figure 4B). The Principle coordination analysis (PCoA) illustrated the bacterial community structures dissimilarities (Adonis, *p* = 0.001, R = 0.463 PERMANOVA) between CK and ST treatments (Figure 4C). Moreover, network analysis identified more connections in the CK than in the ST (Figure 4D; Supplementary Table S5).

By comparing the βNTI and RCbray metrics, respectively, we further investigated whether and how community assembly processes differ between CK and ST. We found that variable selection (βNTI >2) dominates phylogenetic turnover in ST, whereas stochastic processes (|βNTI| < 2) dominate phylogenetic turnover in CK (Figure 4E). The Mantel tests revealed a significant correlation (R = 0.682, *p* = 0.001) between the ST and the composition of the rhizosphere microbiome, whereas no significant correlation (R = 0.375, *p* = 0.194) was found between the CK and the rhizosphere microbiome (Figure 4F). Furthermore, the presence of *Rhizobium*, a keystone taxa in the assembly process of rhizosphere bacterial communities, was more abundant in samples from the ST compared to those from the CK (Figure 4G).

## 4 Discussion

Comprehending the process of rhizosphere microbial assembly is crucial for effectively regulating the composition of the rhizosphere microbial community (Wen et al., 2023; Jiang et al., 2024; Silverstein et al., 2024). Under specific circumstances, the assembly of microbial communities exhibits a high degree of determinism, with distinct communities being influenced by robust environmental selection towards a particular stable state regardless of their initial makeup (Goldford et al., 2018). However, under other circumstances, environmental selection may be less influential, leading to a more unpredictable assembly of communities that rely more heavily on their initial composition (Bittleston et al., 2020). For example, Mendes et al. (2014) found that species abundance in the rhizosphere fits the log-normal distribution model, which indicates the occurrence of niche-based processes (Mendes et al., 2014). Shen et al. (2019) demonstrated that the assembly of the rhizosphere microbiome is influenced by both deterministic and stochastic mechanisms, potentially playing a role in disease suppression (Shen et al., 2019). This finding in our research aligns with prior studies indicating that the assembly of the rhizosphere microbiome is predominantly influenced by deterministic processes (Figure 1).

What’s more, many studies demonstrated that the rhizosphere microbial community could be shaped by plant host habitats, soil factors, phenotypic traits and rhizosphere metabolites (Qu et al., 2020; Wen et al., 2022; Yuan et al., 2022; Zhu et al., 2024). Such as, Wu et al. (2023) observed a transition in the bacterial community assembly process from homogenizing dispersal to variable selection, which was found to be strongly influenced by soil factors such as total phosphorus and carbon-nitrogen ratio (Wu et al., 2023). However, Bi et al. (2022) established that the metabolites present in the rhizosphere exhibited a more pronounced correlation with the structure of rhizosphere bacterial and fungal communities compared to edaphic factors. This finding aligns with the results of the current study, indicating that root exudates plays a significant role in shaping the assembly process of the microbial community (Figure 2).

Specific bioactive metabolites have the potential to influence the composition of the microbial community within the rhizosphere (Bi et al., 2022). A recent study conducted by Wen et al. (2022) demonstrated the deterministic assembly process linked to diseased rhizosphere microbiomes, which exhibited a significant correlation with five specific metabolites: tocopherol acetate, citrulline, galactitol, octadecylglycerol, and behenic acid (Wen et al., 2022). Similarly, Jiang et al. (2024) confirmed that the exogenous administration of a metabolic blend containing essential components enriched through intercropping (soyasapogenol B, 6-hydroxynicotinic acid, lycorine, shikimic acid, and phosphocreatine) notably improved root activity, nutrient levels, and biomass production of maize in indigenous soil (Jiang et al., 2024).

This research enhances comprehension regarding the impact of root exudates on the process of bacterial community assembly. Specifically, our findings indicate that seven compounds, including glycerol, sorbitol, phytol, 1,2,4-benzenetriol, succinate-semialdehyde, alpha-ketoglutaric acid, and D-glyceric acid, play a significant role in driving the assembly of rhizosphere communities, with these compounds primarily belongs to sugars and organic acids (Figure 3). Sugars and organic acids are one of the major components of rhizosphere metabolites (Wen et al., 2020; Liu et al., 2023; Wen et al., 2023). This observation of the importance of sugars and organic acids in plant-microbe interactions has been noted previously (Kamilova et al., 2006; Lebeis et al., 2015; Bezrutczyk et al., 2018). For instance, sugars, frequently utilized as carbon sources for microbial cultures (Chaudhry and Nautiyal, 2011), were discovered to enhance the symbiotic relationships between plants and microorganisms, thereby facilitating adaptation to challenging environmental conditions (Xu et al., 2018; Korenblum et al., 2022). The presence of some organic acids (e.g., malic and fumaric acids) exuded by banana roots plays a vital role in facilitating the colonization of *Bacillus amyloliquefaciens* NJN-6 on the host roots, thereby providing protection against *Fusarium oxysporum* f. sp. *cubense* and promoting the growth of plants (Yuan et al., 2015). Overall, based on these findings, the beneficial effects of specific rhizosphere metabolites (e.g., sugar and organic acids) on plants may be attributed to their role in facilitating the interaction between plant roots and microbes, thus suggesting their potential utility as soil prebiotics (Macias-Benitez et al., 2020).

**Figure 5:**
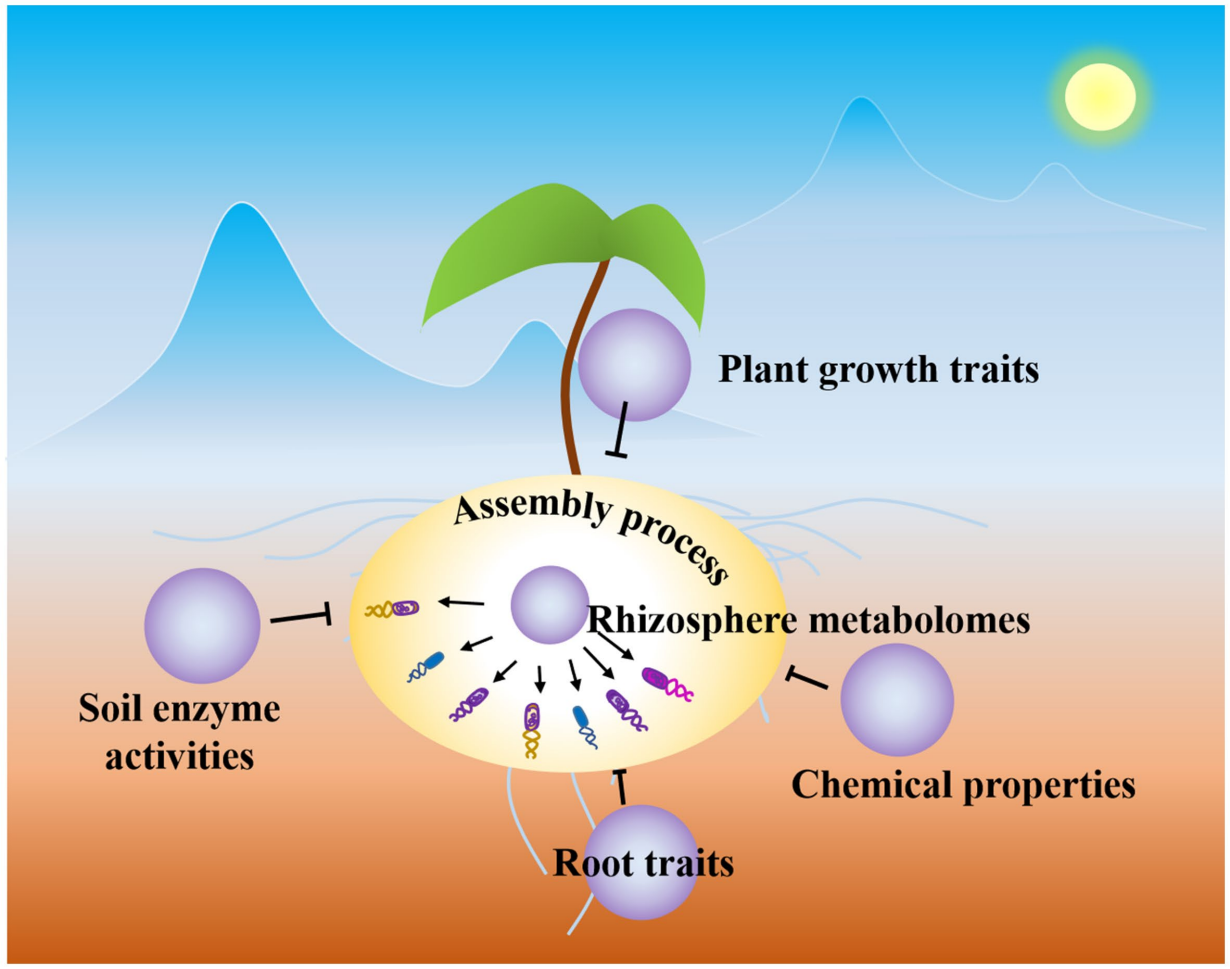
Schematic diagram of rhizosphere microbial community assembly process. Rhizosphere metabolites drive the rhizosphere microbial community assembly process.

Keystone species possess the ability to exert a disproportionate and consequential influence on ecosystems (Berry and Widder, 2014), with their behavior and population levels playing a crucial role in maintaining community stability and long-term sustainability (Mouquet et al., 2013; Wu et al., 2023; Wang X.-W. et al., 2024). Wu et al. (2023) revealed that the keystone species of the phyla Acidobacteriota and Chloroflexi shifted the rhizosphere bacterial community construction process. However, in our research, we discovered that *Rhizobium* may have a significant impact on the assembly of rhizosphere communities (Figure 4). Previous studies have indicated that Rhizobiales, a bacterial order, is one of the most prevalent groups in the rhizosphere (Hacquard et al., 2015; Yeoh et al., 2017). Additionally, it has been suggested that *Rhizobium* plays a crucial role in plant growth and may contribute to microbial homeostasis in healthy roots (Garrido-Oter et al., 2018). For example, Zhong et al. (2019) showed that *Rhizobium* stimulated the proliferation of potentially beneficial microbes, increasing the connections in the rhizobacterial network and altering the hub microbes. Moreover, our study revealed that *Rhizobium*, a potentially keystone taxa, was enriched by seven small molecule metabolites (Figure 4), leading to alterations in microbial communities akin to the rhizosphere assembly process (Figures 4, 5). Core microbes, which play a significant role in community assembly, were found to be crucial for the structure of soil microbial communities. However, the current focus remains on understanding the regulation of these core microbes.

In addition, our findings demonstrate that rhizosphere metabolites play a crucial role in the assembly process of the rhizosphere bacterial community, serving as a mechanism through which small molecule metabolites can be introduced to alter the microbial community by influencing the keystone taxa. However, although our metabolomic studies are conducted under specific conditions and at specific time points, it would be valuable to investigate the relationship between changes in bacterial community structure and metabolites in soil over varying periods of time and geographical locations. Furthermore, it was challenging to ascertain the individual functions of the numerous compounds present in root exudate on the microbial community, as well as to initiate validation experiments with rhizosphere metabolites due to the inability to artificially culture the vast number of microbes involved. Herein, we hope that the function of the microbial community associated with rhizosphere metabolites could be given more attention in future research.

## 5 Conclusion

Our research indicates that rhizosphere metabolites are significantly more influential in the assembly of the rhizosphere microbiome than chemical properties, enzyme activities, and root traits. Specifically, seven compounds present in root exudate, including glycerol, sorbitol, phytol, 1,2,4-benzenetriol, succinate semialdehyde, alpha-ketoglutaric acid, and D-glyceric acid, have been identified as key drivers in this assembly process. Additionally, our findings suggest that *Rhizobium* may serve as a crucial mediator in modulating the composition of the rhizosphere microbial community in response to these small molecule metabolites. The successful regulation of microbial community assembly processes through the manipulation of small-molecule metabolites has been demonstrated. Additionally, an effective method for regulating microbial community assembly processes through the supplementation of small molecule metabolites has been confirmed.

## Data Availability

The datasets presented in this study can be found in online repositories. The names of the repository/repositories and accession number(s) can be found in the article/Supplementary material.
