# Supplementary material for: Small molecule metabolites drive plant rhizosphere microbial community assembly patterns
Source: Front Microbiol. 2025 Feb 11;16:1503537. doi: 10.3389/fmicb.2025.1503537 (PMC11854121; doi:10.3389/fmicb.2025.1503537)
Supplement: Supplementary file 1 [file Data_Sheet_1.docx]

**Supplementary materials**

**Supplementary Method S1.** **Plant samples collection and traits measurement**

On 20^th^ May 2020, cucumber height (cm) was measured from the stem base to the highest growth point (the tallest apical bud). For stem thickness (D, cm), 1 cm below the cotyledon node of the cucumber was marked in black to indicate the location for measurement. The diameter (D) was then taken using a Vernier caliper. The relative growth rate (RGR) was used to evaluate the effect of different fertilizer on the growth of cucumbers. The calculation formula (Eq. (1)) was as follows:

RGR = ln (Δd^2^ × ΔH) / t (1) where Δd is the difference between the final and initial stem thickness, ΔH is the difference between the final and initial height, and t is the measured number of days between the initial and final growth (Baltzer and Thomas 2007).

Ten mature leaves were randomly selected, placed in ziplock bags, and promptly transported to the laboratory for measurement of leaf area (LA) using a leaf area meter (CI-202, Walz, Camas, USA). The residual leaves and stems from each plant were gathered and subsequently placed in No. 11 ziplock bags for drying in an oven at 105°C for 30 minutes, followed by further drying at 65°C for 72 hours. The resulting weights were recorded as the biomass of the leaves and stems (g), respectively.

The complete root system was obtained using the shovelomics method (Trachsel et al. 2011). The pots were opened and a sterilized tweezer was utilized to carefully extract the roots from the soil. We separated the entire root system from the soil by gently shaking and allowing little amounts of soil to adhere to the roots. In order to assess the impact of different types of fertilizer on root system architecture (RSA), a sample of ten cucumber roots was randomly selected, each measuring approximately 20 cm in length from the stem base. We cut the selected roots, rinsed using tap water to remove soil from the surface, and then rinsed three more times using deionized water to ensure no soil particles remained. The roots were wiped dry using absorbent papers to remove surface moisture and were measured for fresh weight. These were then temporarily stored in ziplock bags for RSA.

The isolated roots were carefully positioned horizontally on a plastic plate lined with black photography cloth using a pair of tweezers to separate twines and overlapping roots. Subsequently, the samples were photographed with a Canon EOS 750D Digital SLR Camera (EF-S 18-135 mm f / 3.5 - 5.6 IS STM lens, 24.2 million pixels) positioned at a vertical height of 60 cm. All images were saved and analyzed using SmartRoot software (Lobet et al. 2011) for subsequent RSA analysis to determine average lateral root diameter (LRAD), total lateral root number (TLRN), total lateral root length (TLRL), mean inter-branch density (MID), maximum order of lateral roots (MaxO), and branching density (BI, ration of branches number to root length, cm^-1^). After photo documentation, the roots were placed separately in bubble envelopes, dried at 70 °C for 72 h, and weighed. Finally, the remaining roots were also collected respectively, dried at 65℃ for 72 h, and weighed for the fine root biomass (RFB, g). In addition, the following quantities were calculated: root-to-shoot ratio (R/S) was the total roots dry weight divided by the total dry weight of leaves plus stems.

**Supplementary Method S2.** **Rhizosphere soil chemical traits and extracellular enzyme activities measurement**

An acidometer (MT-5000, Shanghai, China) was used to measure soil pH, while a flow injection auto-analyzer (AA3, Seal Co., Germany) was used to determine soil ammonium (NH_4_^+^-N) and nitrate (NO_3_^-^-N) concentrations. Total soil N (TN) concentrations were determined using an elemental analyzer (Vario Macro, Elementar Analysensysteme, Germany). The soil organic matter (SOM) was determined by the chromic acid titration method based on the methodology. Sodium bicarbonate was used to extract soil available P (AP) and thenquantified using the molybdenum blue method (Bao 2000). Ammonium acetate was used to extract soil available K (AK) and exchangeable Ca and Mg were quantified using flame photometry. Available Fe, Mn, Cu, and Zn were leached with hydrochloric acid (Bao 2000), then quantified using ICP-AES (Thermo Jarrell Ash Ltd., Franklin, MA, USA).

The MUB-linked model substrates method was used to determine the extracellular enzyme activities (EEAs) related to carbon, nitrogen, and phosphorus cycles (Table S1). These included α-glucosidase (αG), β-glucosidase (βG), cellobiose hydrolysis enzyme (CBH), L-leucine amino peptidase (LAP), acid phosphatase (ACP), and β-1,4-N-acetylglucosaminidase (NAG). Phenol oxidase (PhO) and peroxidase (PeO) activities were measured spectrophotometrically in clear 96-well microplates using L-3,4-dihydroxy-phenylalanine (DOPA) as the substrate (DeForest 2009). Enzyme activity was expressed as nmol h^-1^ g^-1^ soil. The response of soil physiochemical properties, enzyme activities, root traits and plant growth traits to different fertilizer managements is detailed in Table S4.

**Supplementary Method S3.** **16S rRNA gene amplification and amplicon sequencing**

The DNA quality and quantity were measured via a 1.2% agarose gel and a NanoDrop 1000 spectrophotometer (Thermo Scientific, USA). For amplification, the 50 μL reaction mixtures contained 25 μL 2× Premix Taq (Takara Biotechnology, Dalian Co. Ltd., China), 1 μL each primer (10 μM), 3 μL DNA (20 ng μL^-1^) and 25 μL of sterilized ultrapure water. PCR amplification was performed by using a BioRad S1000 (Bio-Rad Laboratory, CA) with the following cycles: 95°C for 5 min, then 30 cycles of 94°C for 30 s, 52°C for 30 s, 72°C for 30 s with a final extension at 72 °C for 10 min. Products were run on a 1% agarose gel, and those with clear bands between 290-310 bp were combined for sequencing (Wen et al. 2020, Wen et al. 2022). PCR products were mixed in equidensity ratios according to the GeneTools Analysis Software (Version 4.03.05.0, SynGene), and the mixture was purified with an EZNA Gel Extraction Kit (Omega, USA). Sequencing libraries were generated using the NEBNext® Ultra™ DNA Library Prep Kit for Illumina® (New England Biolabs, USA) following the manufacturer's recommendations. Index codes were added, and library quality was assessed with a Qubit® 2.0 Fluorometer (Thermo Scientific) and an Agilent BiCAnalyzer 2100 system (Wen et al. 2020). Finally, the library was sequenced on an Illumina MiSeq sequencing platform using PE250 chemicals at the Genomics Core of Michigan State University. Clean reads were quality filtered according to Trimmomatic (V0.33, http://www.usadellab.org/cms/?page=trimmomatic), and sequences were assigned to each sample based on its unique barcode. All of the clean reads were processed using the pipelines detailed above (Wen et al. 2020, Wen et al. 2022).

For sequencing data analyses, Usearch (V. 10.1) and Vsearch (V. 0.6.3) were used for the main procession (Wen et al. 2020). First, “vsearch --fastq_mergepairs” script was used for pair-end sequence merge; “vsearch --fastx_filter” script was used for primers cut; “vsearch --derep_fulllength” script was used for finding unique read sequences; “usearch -cluster_otus” script was used for cluster ASVs with 97% similarity. “Vsearch --usearch_global” script was used for creating ASV table. “vsearch --sintax” script and RDP tax database were together used for annotation (Wen et al. 2020).

**Supplementary Method S4.** **Detailed procedures for low molecular weight molecules extraction**

One part of the soil samples was homogenized in a ball mill for 4 min at 45Hz in 0.5 mL methanol solution (*V*_methanol_: *V*_H2O_ = 3:1), then ultrasound treated for 5 min (incubated in ice water) 5 times. The supernatant (0.4 mL) was transferred into a fresh centrifuge tube after being centrifuged for 15 min at 10000×g at 4 °C. The residue was extracted with 0.5 mL ethyl acetate using the method mentioned above: then 0.4 mL of ethyl acetate extraction was transferred to the methanol extraction. The other part of the soil sample was extracted by ethyl acetate followed by methanol using the method reported above. Overall of 1.6 mL (0.4 x 4) solution was obtained from one soil sample, and 40 μL were taken and pooled as QC sample, and 1.2 mL were transferred into a fresh 2 mL GC/MS glass vial and dried with nitrogen gas. Then 20 μL methoxyamination hydrochloride (20 mg mL^−1^ in pyridine) were added to the dried sample and incubated for 30 min at 80 ℃before being treated with 30 μL of the BSTFA (bis(trimethylsilyl) trifluoroacetamide) regent (1% TMCS (Trimethylchlorosilane),v/v). Finally, the mixture was incubated for 1.5h at 70℃.

**Supplementary Method S5.** **Detailed procedures for the incubation experiment**

The seven compounds demonstrated a significant correlation with the assembly process of the rhizosphere microbial community. The soil utilized in the study was consistent with the previously mentioned soil. Solutions in water were prepared such that they contained each of the selected compounds in equal amounts (1.4 mM glycerol, 1.4 mM Sorbitol, 1.4 mM Phytol, 1.4 mM 1,2,4-Benzenetriol, 1.4 mM succinate semialdehyde, 1.4 mM alpha-ketoglutaric acid, and1.4 mM D-Glyceric acid) and at a final total concentration of about 10 mM. Prior to the addition of the compound mixture, 50 g of soil were placed into each plate. Subsequently, the plates were preincubated in a growth chamber at 30 °C for one week to facilitate acclimation of the soil microbiome and to enable differentiation between seedling rhizospheres and the bulk soil. Each well received 5 ml of compound mixture solution twice a week for 4 ½ weeks (2 total applications) in a growth chamber at 30 °C (Yuan et al. 2018). Sterile ultrapure water was added to each well as a control (CK). Each treatment consisted of 6 plates. All the plates were randomly arranged during the incubation period.

**Supplementary Method S6.** **Rhizosphere community assembly process analysis**

Firstly, for this analysis, we used modified R code from (Burns 2015). We estimated each ASV’s abundance in the metacommunity (pi) by averaging its relative abundance across all samples. The detection threshold (d) is equal to 1/N. The model was generated using the function pbeta from package stats (R Core Team 2018) and fit to data using function nlsLM from package minipack.lm (Elzhov et al. 2016). The function binconf from package Hmisc (Akwo et al. 2018) was used to calculate a 95% prediction interval.

Second, we estimated the influence of deterministic processes on community assembly by calculating the β-nearest taxon index (βNTI) between pairs of samples as described in (Stegen et al. 2012). This null modeling approach compares the observed phylogenetic turnover in ASVs between samples relative to a stochastic model. We first determined the observed abundance weighted β-mean-nearest taxon distance (βMNTD) using function comdistnt from package Picante (Kembel et al. 2010). Then, we generated the βMNTD null model by randomly shuffling the tips of the phylogenetic tree and recalculating the pairwise βMNTD values. We repeated this reshuffling step 999 times to generate a null distribution for each pair. Then, we calculated βNTI for each pairwise samples comparison. βNTI was calculated based on the entire metacommunity. Samples pairs with |βNTI| > 2 are expected to result from deterministic processes, as previously described (Stegen et al., 2012). More specifically, βNTI 2 indicates that variable selection is the dominant assembly process between samples because phylogenetic turnover is more significant than expected by chance (Sugawara et al. 2013). Variable selection occurs when biotic or abiotic factors impose different selection pressures between samples, driving divergence in community composition. A βNTI < -2 indicates that homogenous selection is the dominant assembly process between samples because phylogenetic turnover is significantly less than expected by chance (Zeisel et al. 2011). Homogeneous selection occurs when the selection pressures are similar between samples, driving convergence in community composition. Samples pairs with |βNTI| < 2 indicate that selection pressure is weak and community assembly is likely governed by stochastic processes (Kembel et al., 2010). If factors influence community assembly processes, we expect phylogenetic turnover (as measured by βNTI) will correlate with an environmental dissimilarity. Mantel tests (package vegan) were performed to evaluate whether βNTI correlated with differences (expressed as Euclidian distance) in each of the factors distance.

**Figure S1** Metabolites are grouped according to their chemical properties and plotting with stacked column charts.

**
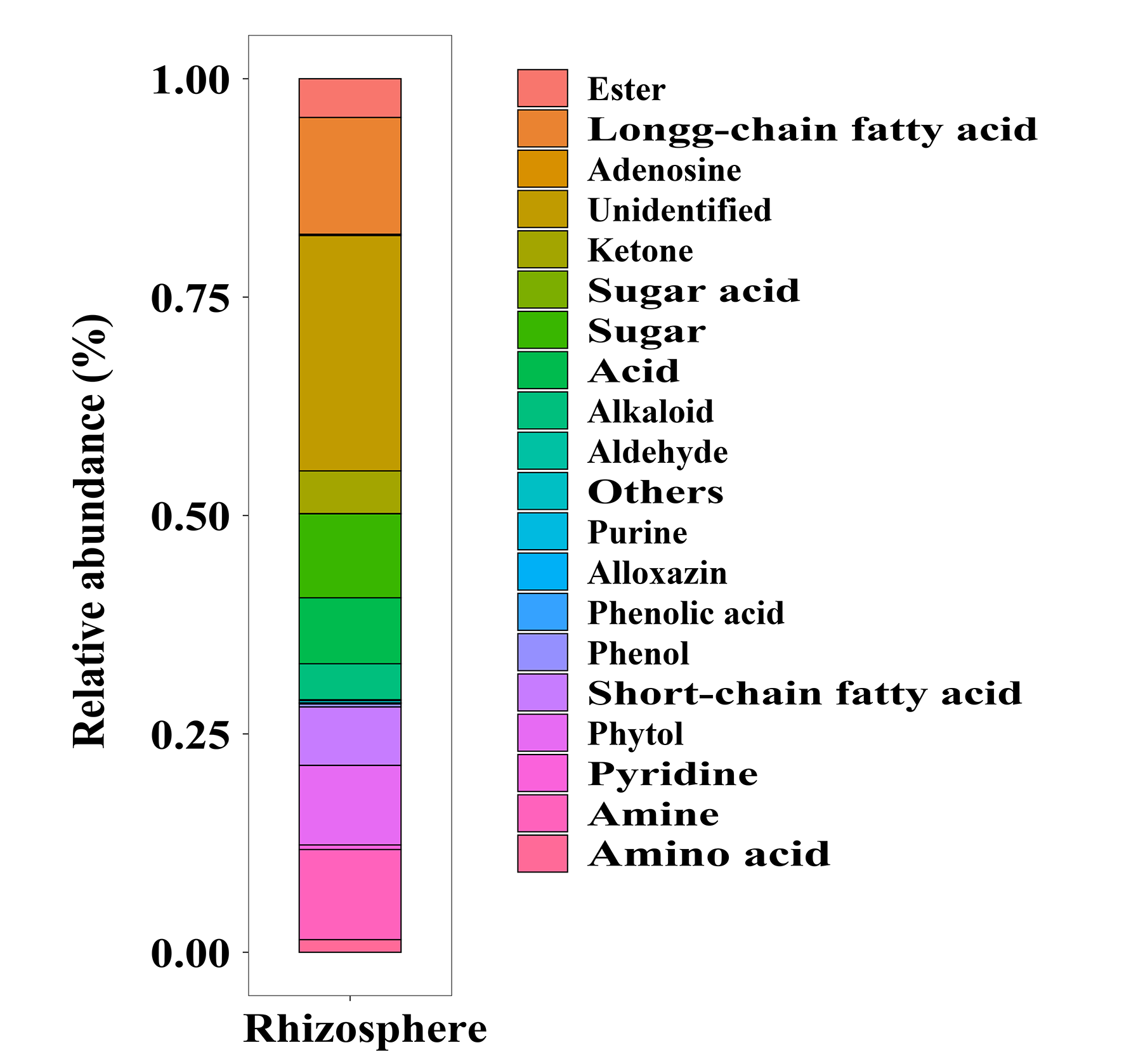
**

**Figure S2** Mantel test correlation of bacterial communities and assembly process and different group factors using the Mantel test (999 permutations). All abbreviated indicators are detailed in Fig. 2.

**
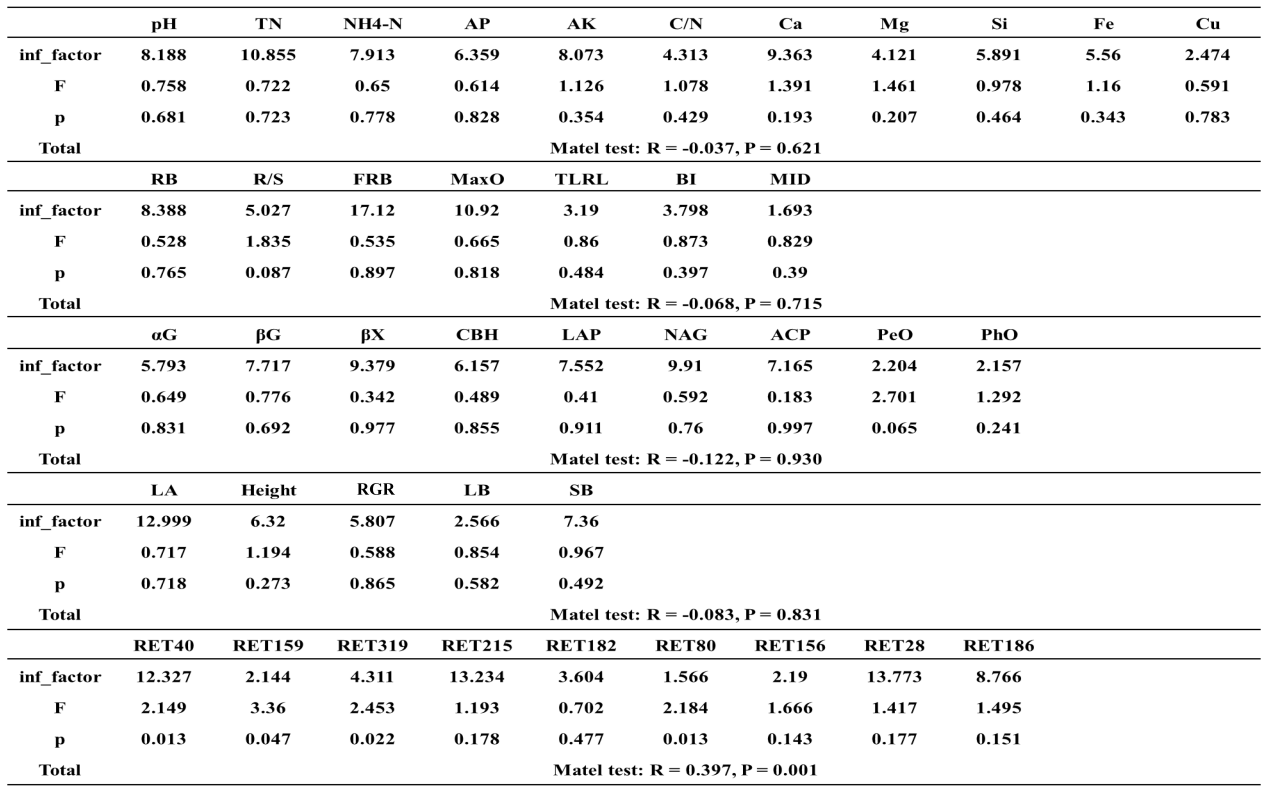
**

**Figure S3** Mantel correlation of βNTI values and soil chemical properties, enzyme activities and root traits.


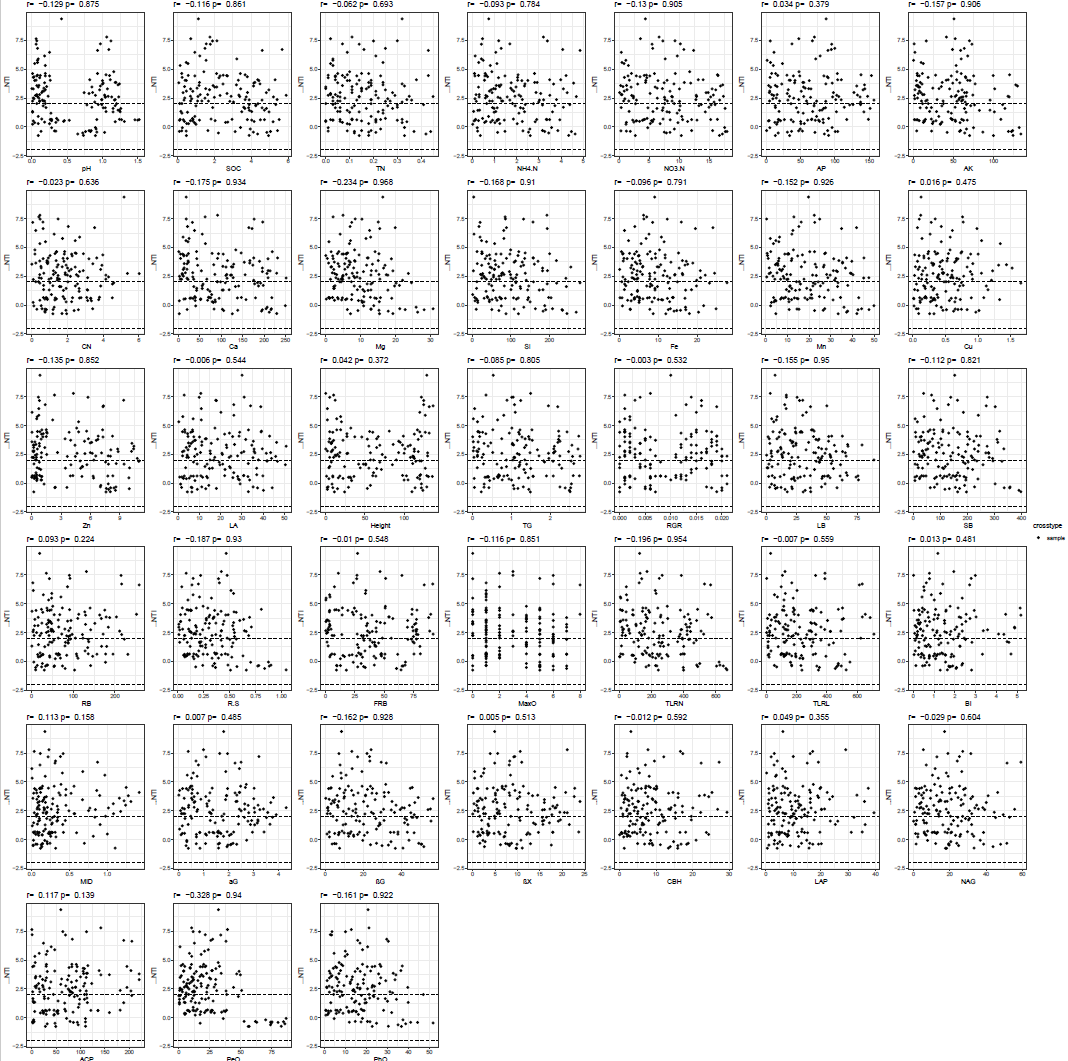


**Figure S4** Mantel correlation of βNTI values and Phytol compounds.


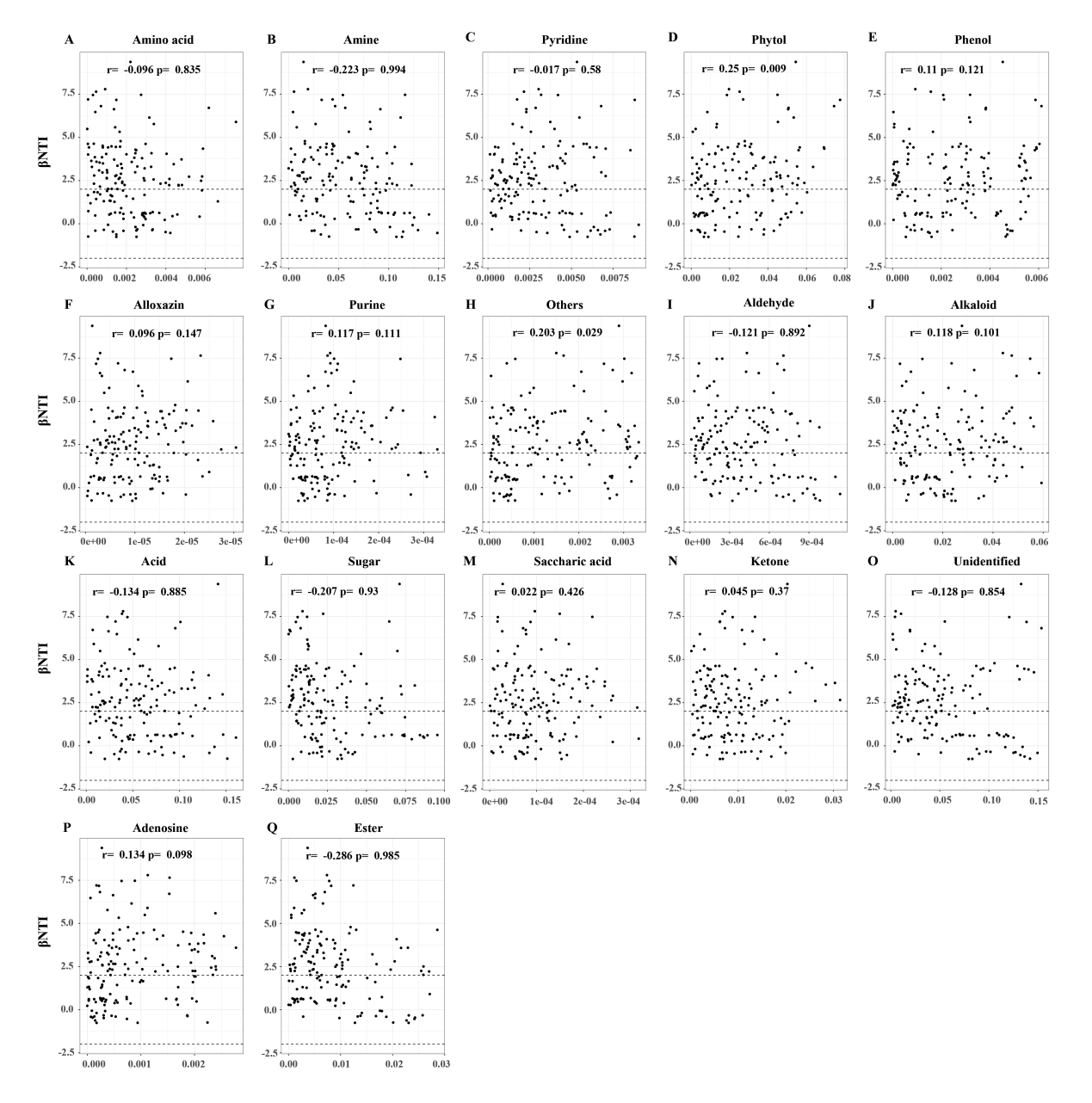


**Figure S5** Mantel correlation of βNTI values and seven compounds belong to phytol compounds.

**
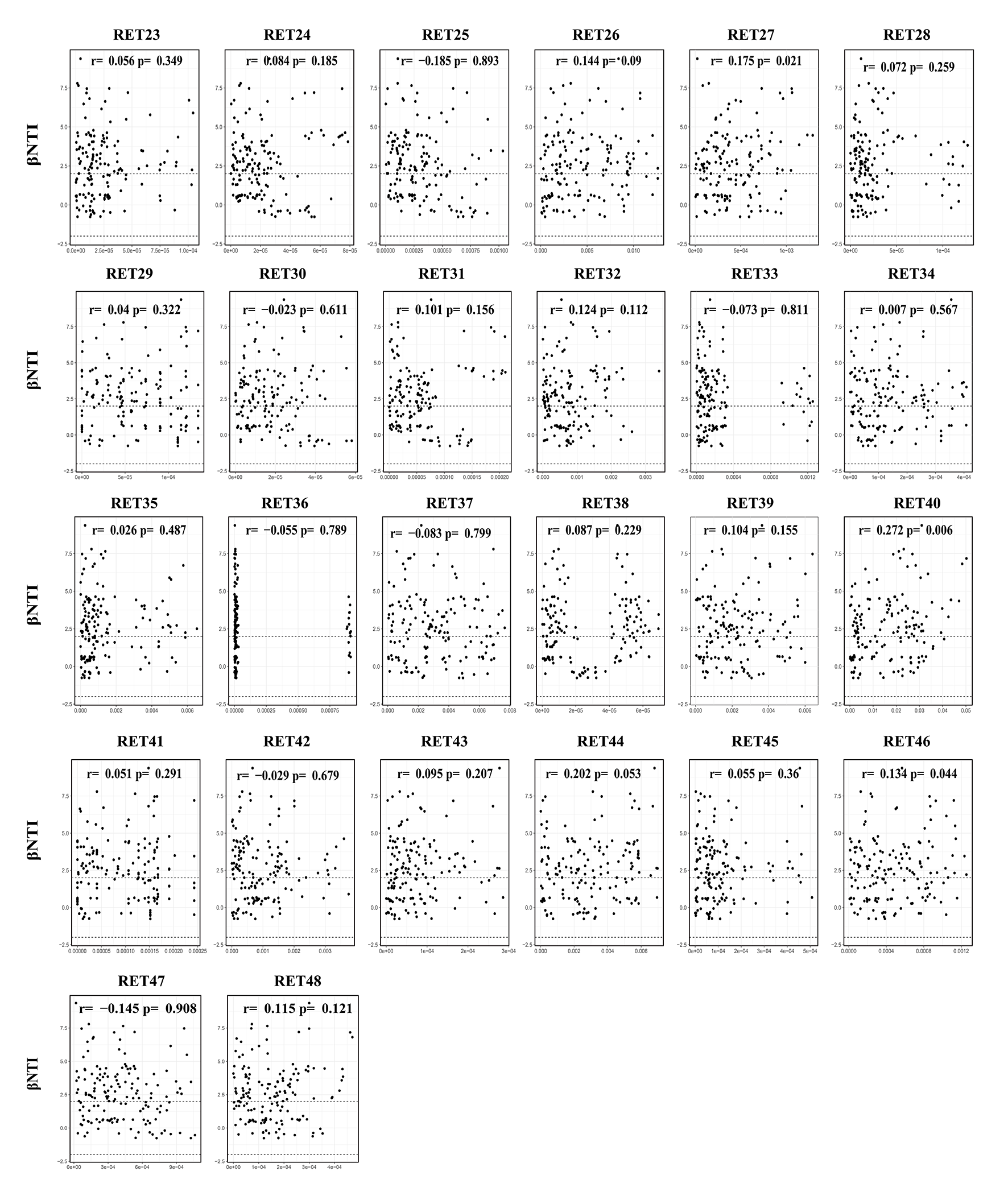
**

**Figure S6** Mantel correlation of βNTI values and compounds with the coefficient of variation greater than 50%.

**
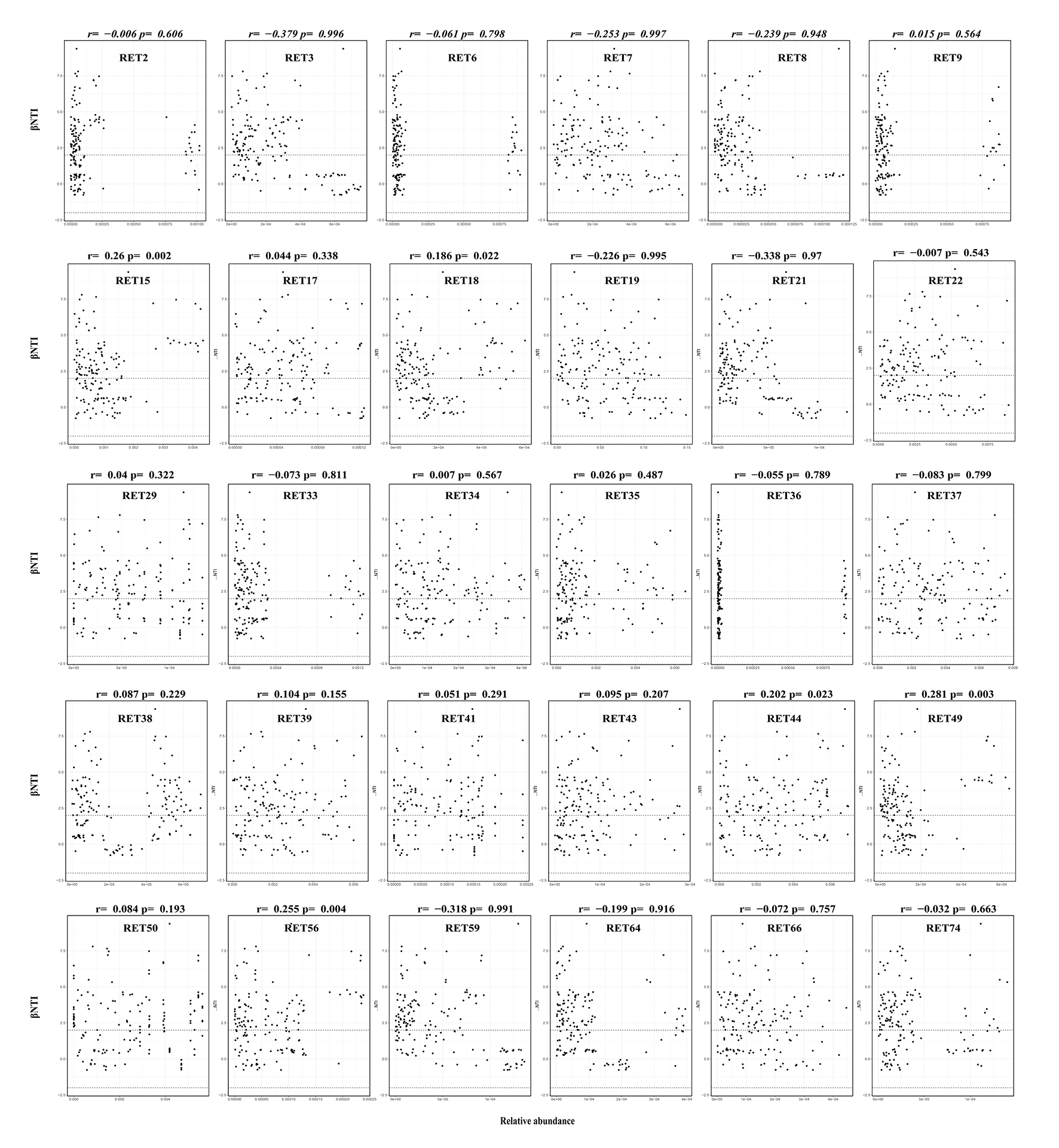
**

**
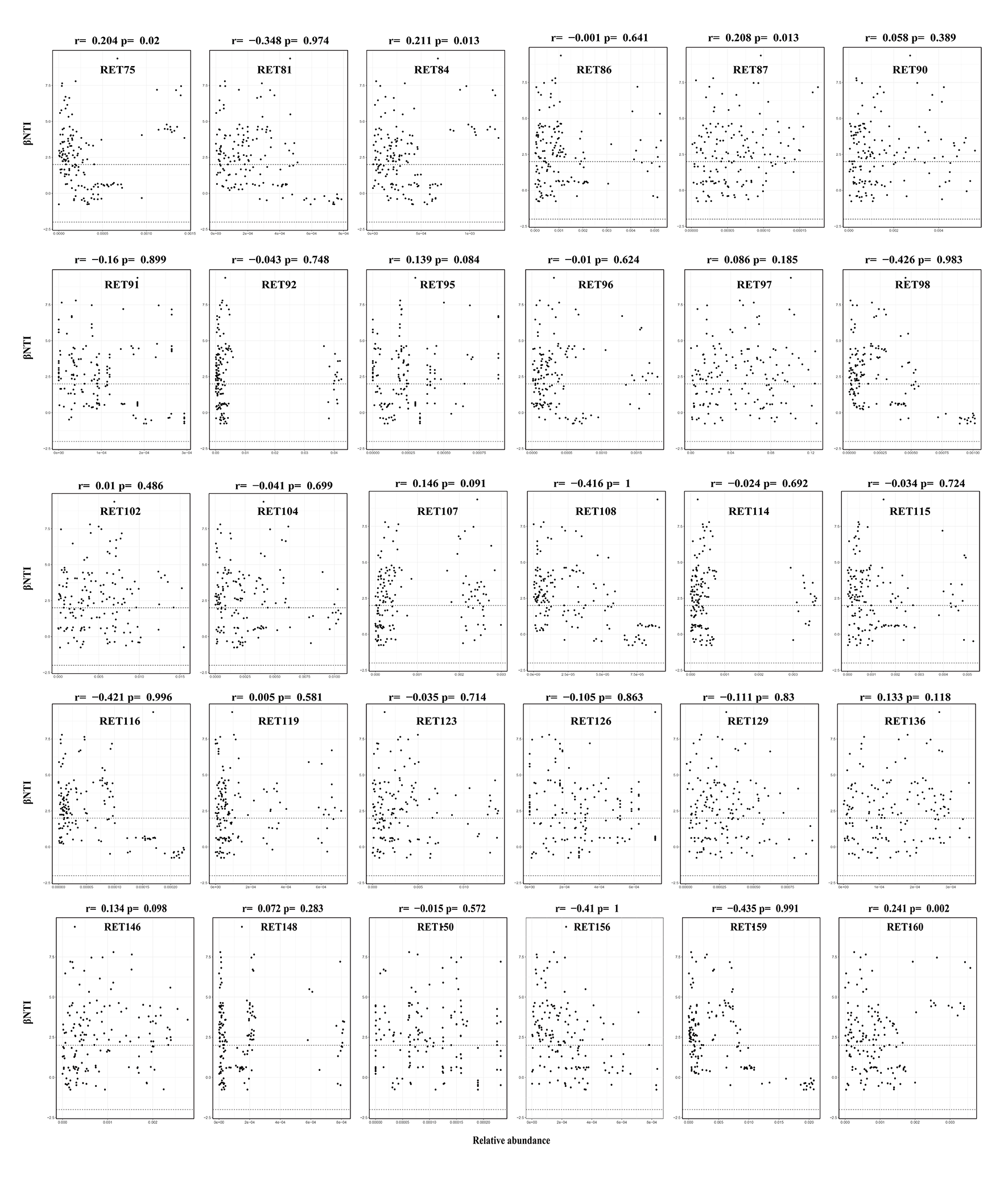
**

**
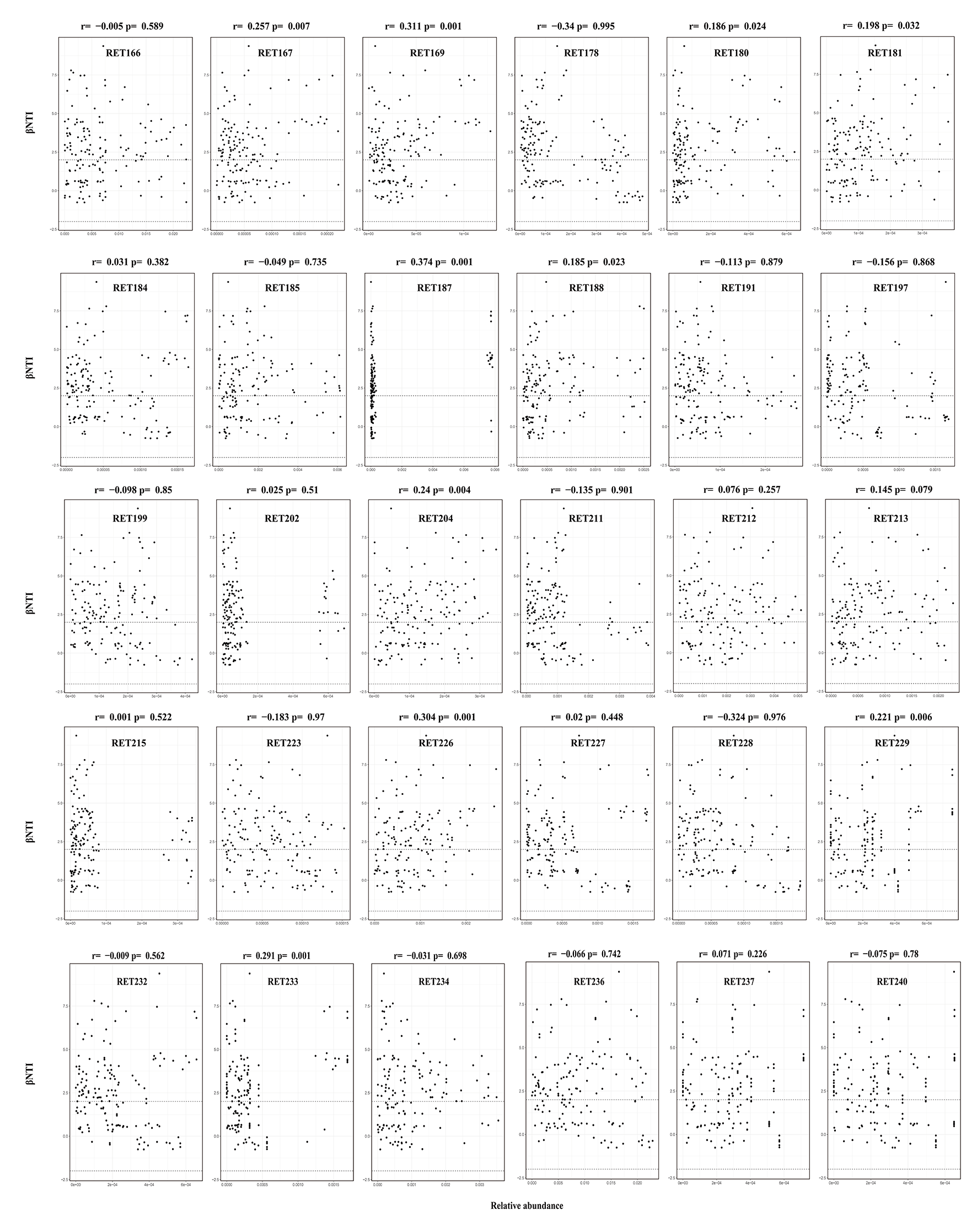
**

**
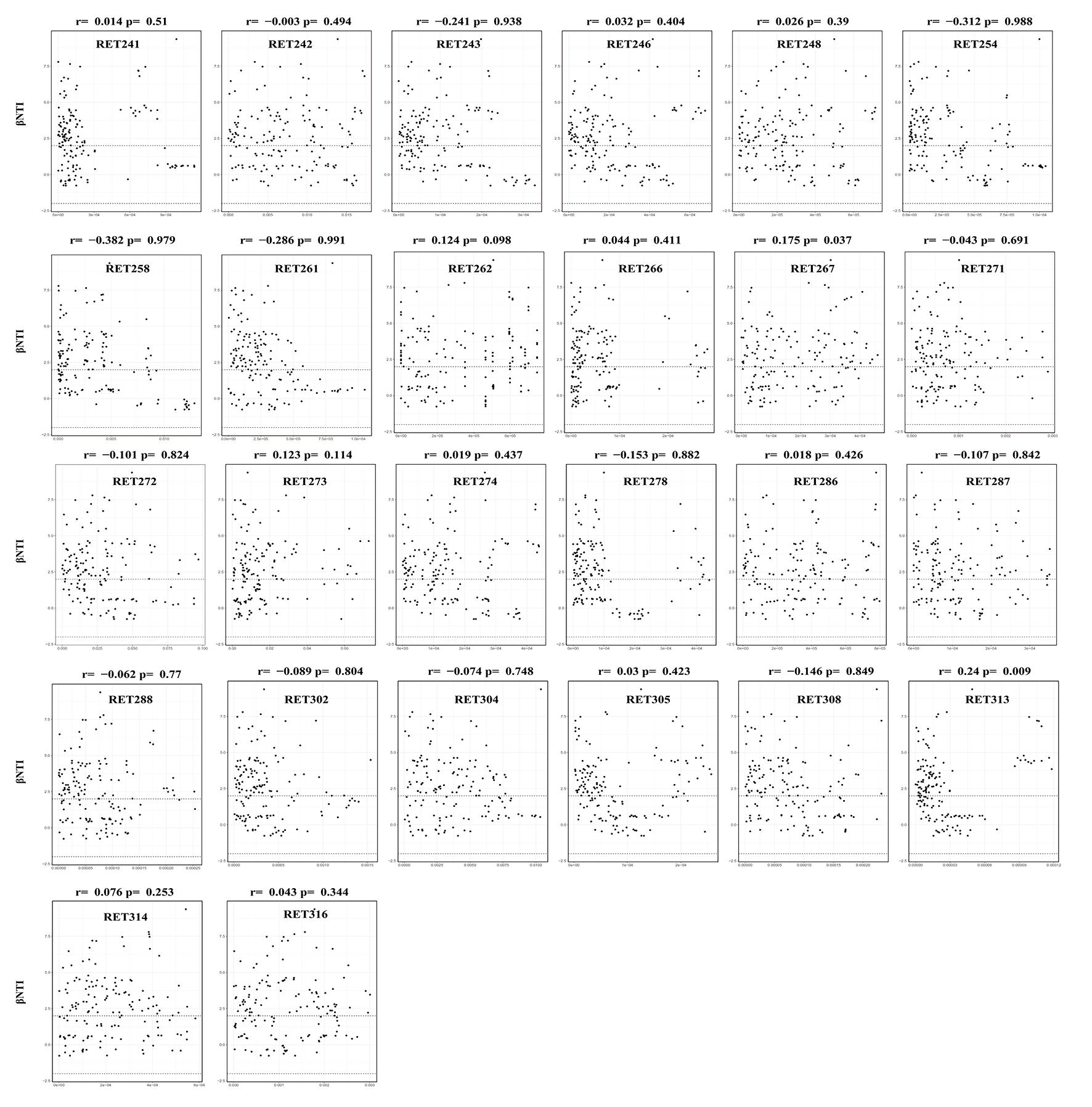
**

**Figure S7** Mantel correlation of βNTI values and taxa at Genus levels.


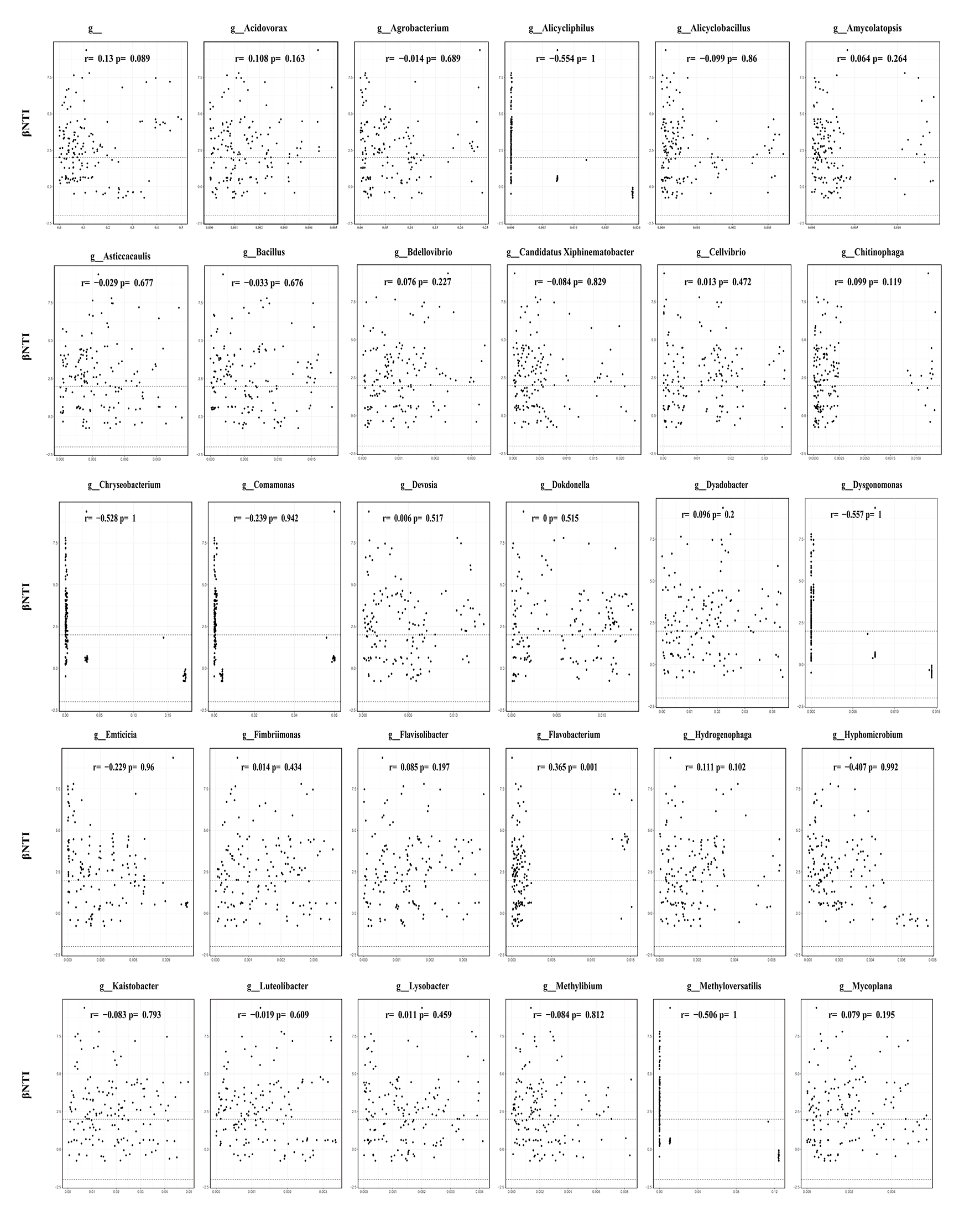


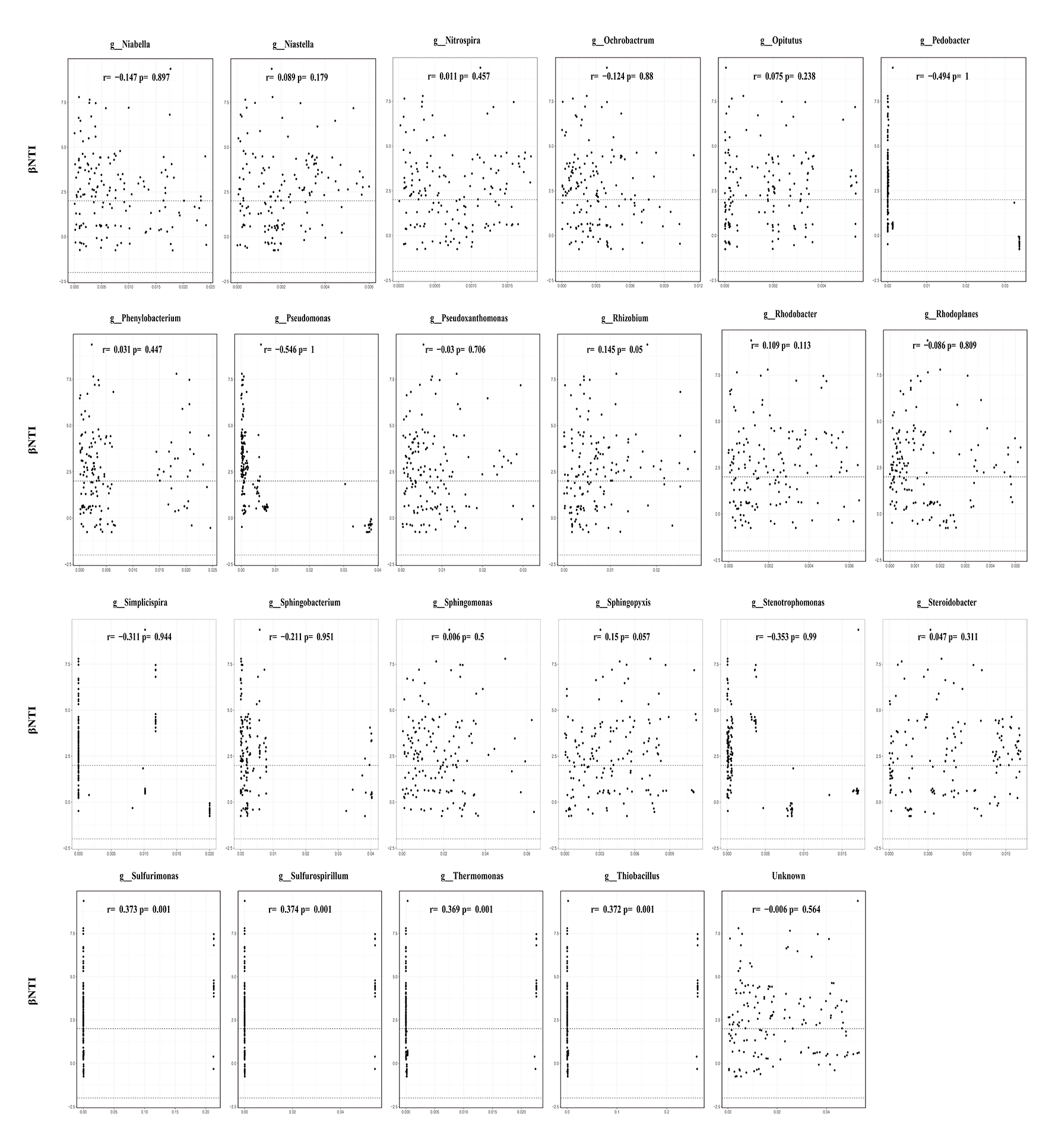


**Table S1.** Soil enzymes with corresponding commission number (EC), corresponding substrate, and the abbreviation used in this study.

| Cor. Cycle^1^ | Enzyme | Abbreviation | Substrate | EC |
| --- | --- | --- | --- | --- |
| C-cycling  N-cycling | Peroxidas | PeO | L-DOPA^3^ | 1.11.1.7 |
|  | Phenol oxidase | PhO | L-DOPA | 1.10.3.2 |
|  | α-1,4-Glucosidase | αG | 4-MUB^2^-α-D-glucoside | 3.2.1.20 |
|  | β-1,4-Glucosidase | βG | 4-MUB-β-D-glucoside | 3.2.1.21 |
|  | β-1,4-xylosidase | βX | 4-MUB-β-D-xyloside | 3.1.1.37 |
|  | β-D-Cellobiohydrolase | CBH | 4-MUB-β-D-cellobioside | 3.2.1.91 |
|  | Leucine amino peptidase | LAP | L-Leucine-7-amino-4-methylcoumarin | 3.4.11.1 |
|  | β-1,4-N-Acetyl-glucosaminidase | NAG | 4-MUB-N-acetyl-β-D-glucosaminide | 3.2.1.30 |
| P-cycling | Acid phosphomonoesterase | ACP | 4-MUB-phosphate | 3.1.3.2 |

^1^ The enzymes involved in this cycling process

^2^ 4-MUB, 4-methylumbelliferyl
^3^ L-DOPA, L-3,4-dihydroxyphenylalanine

**Table S2** Phylogenetic signals analyses of soil chemical properties, enzyme activities, and plant growth traits to rhizosphere microbial community.

|  | K | PIC.variance.obs | PIC.variance.rnd.mean | PIC.variance.P | PIC.variance.Z |
| --- | --- | --- | --- | --- | --- |
| pH | 0.104 | 1.019 | 1.193 | 0.241 | -0.764 |
| SOC | 0.070 | 0.801 | 0.634 | 0.850 | 1.015 |
| TN | 0.085 | 0.514 | 0.492 | 0.607 | 0.147 |
| NH4-N | 0.108 | 0.575 | 0.708 | 0.222 | -0.782 |
| NO3-N | 0.108 | 1.064 | 1.314 | 0.130 | -1.068 |
| AP | 0.108 | 0.115 | 0.143 | 0.426 | -0.340 |
| AK | 0.102 | 0.549 | 0.634 | 0.334 | -0.503 |
| CN | 0.106 | 0.532 | 0.633 | 0.294 | -0.588 |
| Ca | 0.086 | 1.938 | 1.884 | 0.604 | 0.198 |
| Si | 0.091 | 2.026 | 2.076 | 0.451 | -0.173 |
| Fe | 0.087 | 0.572 | 0.567 | 0.558 | 0.028 |
| Mn | 0.094 | 0.067 | 0.070 | 0.541 | -0.067 |
| Cu | 0.072 | 0.873 | 0.704 | 0.843 | 0.950 |
| Zn | 0.093 | 0.270 | 0.285 | 0.514 | -0.142 |
| LA | 0.132 | 0.378 | 0.561 | 0.098 | -1.193 |
| TG | 0.110 | 1.058 | 1.329 | 0.111 | -1.159 |
| RGR | 0.139 | 0.488 | 0.768 | **0.037** | -1.540 |
| LB | 0.146 | 1.645 | 2.218 | **0.021** | -1.828 |
| SB | 0.103 | 1.292 | 1.496 | 0.187 | -0.852 |
| RB | 0.143 | 2.388 | 3.533 | **0.001** | -2.717 |
| R/S | 0.115 | 0.703 | 0.839 | 0.248 | -0.714 |
| FRB | 0.120 | 0.664 | 0.899 | 0.101 | -1.196 |
| MaxO | 0.102 | 3.000 | 3.388 | 0.150 | -1.006 |
| TLRN | 0.134 | 0.415 | 0.631 | 0.062 | -1.324 |
| TLRL | 0.107 | 0.537 | 0.647 | 0.265 | -0.648 |
| BI | 0.087 | 0.722 | 0.705 | 0.561 | 0.102 |
| MID | 0.116 | 0.482 | 0.625 | 0.183 | -0.872 |
| αG | 0.116 | 0.924 | 1.190 | 0.097 | -1.206 |
| βG | 0.093 | 0.803 | 0.839 | 0.448 | -0.199 |
| βX | 0.095 | 0.592 | 0.641 | 0.414 | -0.298 |
| CBH | 0.080 | 0.236 | 0.213 | 0.667 | 0.236 |
| LAP | 0.112 | 0.719 | 0.909 | 0.156 | -0.967 |
| NAG | 0.134 | 0.416 | 0.635 | 0.064 | -1.307 |
| PeO | 0.115 | 0.792 | 0.986 | 0.182 | -0.899 |
| PhO | 0.067 | 1.001 | 0.758 | 0.900 | 1.342 |

**Table S3** phylogenetic signals analyses of rhizosphere metabiotics to rhizosphere microbial community.

|  | K | PIC.variance.obs | PIC.variance.rnd.mean | PIC.variance.P | PIC.variance.Z |
| --- | --- | --- | --- | --- | --- |
| RET40 | 0.121 | 6.936 | 9.560 | **0.001** | -3.539 |
| RET46 | 0.123 | 2.484 | 3.433 | **0.004** | -2.450 |
| RET44 | 0.131 | 5.078 | 6.564 | **0.001** | -2.448 |
| RET49 | 0.085 | 0.878 | 0.833 | 0.635 | 0.238 |
| RET56 | 0.109 | 4.354 | 5.179 | **0.029** | -1.816 |
| RET75 | 0.092 | 0.946 | 0.992 | 0.451 | -0.226 |
| RET84 | 0.083 | 0.979 | 0.902 | 0.671 | 0.407 |
| RET87 | 0.121 | 4.368 | 6.007 | **0.001** | -2.975 |
| RET26 | 0.114 | 4.778 | 6.128 | **0.003** | -2.493 |
| RET27 | 0.069 | 1.905 | 1.479 | 0.940 | 1.610 |

**Table S4** Soil chemical properties, enzyme activities and plant growth traits in rhizosphere soil under the different fertilizer treatments.

| Groups | Treatments | pH | SOC | TN | NH4-N | NO3-N | AP | AK | C/N | Ca |
| --- | --- | --- | --- | --- | --- | --- | --- | --- | --- | --- |
| Soil chemical properties | NF | 5.08±0.04b | 10.18±0.37b | 0.91±0.05c | 18.47±0.22c | 43.87±0.36c | 67.89±2.98c | 79.56±2.43c | 11.41±0.86ab | 220.49±7.81b |
|  | CF | 4.95±0.06b | 10.87±0.53b | 1.03±0.03b | 19.48±0.21b | 50.94±0.94b | 137.83±7.07b | 130.78±6.38b | 10.57±0.44b | 261.78±15.18b |
|  | BIO | 6.06±0.06a | 14.37±0.14a | 1.17±0.03a | 21.80±0.40a | 59.20±0.72a | 187.94±9.22a | 166.97±13.66a | 12.37±0.34a | 397.85±17.83a |
|  | Treatments | Mg | Si | Fe | Mn | Cu | Zn |  |  |  |
|  | NF | 37.92±2.56b | 216.49±16.54b | 42.14±1.73c | 28.41±1.81c | 2.05±0.17a | 2.91±0.13b |  |  |  |
|  | CF | 43.51±2.04b | 252.73±18.00b | 47.93±1.29b | 41.70±2.44b | 2.06±0.09a | 3.71±0.14b |  |  |  |
|  | BIO | 53.09±2.04a | 379.68±19.63a | 58.36±1.86a | 64.39±2.65a | 2.42±0.22a | 5.88±0.54a |  |  |  |
| Soil enzyme activities | Treatments | αG | βG | βX | CBH | LAP | NAG | ACP | PeO | PhO |
|  | NF | 2.02±0.15c | 22.67±1.79c | 12.01±1.27b | 18.69±1.21b | 9.97±0.99c | 35.43±2.84c | 105.06±5.62c | 87.60±4.24a | 98.25±4.36b |
|  | CF | 3.39±0.37b | 38.20±2.73b | 14.96±1.28b | 22.06±1.21b | 17.78±1.44b | 54.46±1.90b | 175.67±8.31b | 97.65±5.91a | 96.06±5.29b |
|  | BIO | 4.91±0.23a | 65.22±3.41a | 27.25±1.95a | 34.03±2.55a | 29.97±3.37a | 70.73±4.58a | 235.94±22.01a | 108.02±12.96a | 116.78±3.79a |
| Root traits | Treatments | TLRN | TLRL | BI | MID | MaxO | FRB | R/S |  |  |
|  | NF | 144.17±10.49c | 297.73±31.40b | 1.50±0.18b | 1.09±0.20a | 3.0±0.37c | 0.40±0.00c | 0.07±0a |  |  |
|  | CF | 325.00±18.92b | 428.64±50.29b | 1.37±0.29b | 0.88±0.05ab | 4.0±0.31b | 0.53±0.01b | 0.05±0b |  |  |
|  | BIO | 618.83±39.35a | 665.14±72.91a | 3.64±0.49a | 0.62±0.04b | 7.0±0.52a | 0.70±0.01a | 0.05±0b |  |  |
| Growth traits | Treatments | LA | Height | SD | RGR | LB | SB |  |  |  |
|  | NF | 152.10±2.17b | 67.17±2.79b | 0.26±0.017c | 0.025±0.002c | 7.95±0.40b | 3.43±0.18c |  |  |  |
|  | CF | 177.58±1.82b | 166.51±11.57a | 0.32±0.016b | 0.047±0.002b | 11.66±1.07a | 5.59±0.19b |  |  |  |
|  | BIO | 190.13±1.75a | 180.33±5.14a | 0.49±0.022a | 0.063±0.001a | 12.61±0.40a | 6.75±0.31a |  |  |  |

Note: SOC, soil organic carbon; TN, total nitrogen; NH4-N, ammonium nitrogen; NO3-N, nitrate-nitrogen; AP, available phosphorus; AK, available potassium; C/N, ratio of soil carbon content to nitrogen content; αG,α-1,4-Glucosidase; βG, β-1,4-Glucosidase; βX, β-1,4-xylosidase; CBH, β-D-Cellobiohydrolase; LAP, leucine amino peptidase; NAG, β-1,4-N-Acetyl-glucosaminidase; ACP, acid phosphomonoesterase; PeO, peroxidase; PhO, phenol oxidase; TLRN, total lateral root number; TLRL, total lateral root length; BI, branching intensity; MID, mean inter-branch distance; MaxO, maximum order of lateral roots; FRB, fine root biomass; R/S, ration of root biomass to shoot biomass; LA, leaf area; SD, steam diameter; RGR, relative growth rate; LB, leaf biomass; SB, steam biomass. Each value represents the mean (n = 6), and the error bars are the standard errors. Significant differences are indicated by different lowercase letters at *P < 0.05* based on the LSD test.

**Table S5** Globe network properties in CK and ST

|  | CK | ST |
| --- | --- | --- |
| Num.edges | 1004 | 846 |
| Num.pos.edges | 541 | 416 |
| Num.neg.edges | 463 | 430 |
| Num.vertices | 149 | 150 |
| Connectance | 0.091 | 0.076 |
| Average.degree | 13.477 | 11.280 |
| Average.path.length | 3.001 | 3.162 |
| Diameter | 5.119 | 5.200 |
| Edge.connectivity | 2 | 2 |
| Clustering.coefficient | 0.569 | 0.529 |
| No.clusters | 1 | 1 |
| Centralization.degree | 0.112 | 0.099 |
| Centralization.betweenness | 0.049 | 0.032 |
| Centralization.closeness | 0.123 | 0.085 |

Akwo, E. A., E. K. Kabagambe, F. E. Harrell, W. J. Blot, J. M. Bachmann, T. J. Wang, D. K. Gupta, and L. Lipworth. 2018. Neighborhood Deprivation Predicts Heart Failure Risk in a Low-Income Population of Blacks and Whites in the Southeastern United States. Circulation: Cardiovascular Quality and Outcomes **11**:e004052.

Baltzer, J. L., and S. C. Thomas. 2007. Determinants of whole-plant light requirements in Bornean rain forest tree saplings. Journal of Ecology **95**:1208-1221.

Bao, S. 2000. Soil agrochemical analysis. China Agricultural Press, Beijing, Chinese.

Burns, D. 2015. How change happens: The implications of complexity and systems thinking for action research. The SAGE handbook of action research:434-445.

DeForest, J. L. 2009. The influence of time, storage temperature, and substrate age on potential soil enzyme activity in acidic forest soils using MUB-linked substrates and l-DOPA. Soil Biology and Biochemistry **41**:1180-1186.

Elzhov, T. V., K. M. Mullen, A.-N. Spiess, B. Bolker, M. K. M. Mullen, and M. Suggests. 2016. Package ‘minpack. lm’. Title R Interface Levenberg-Marquardt Nonlinear Least-Sq. Algorithm Found MINPACK Plus Support Bounds.

Kembel, S. W., P. D. Cowan, M. R. Helmus, W. K. Cornwell, H. Morlon, D. D. Ackerly, S. P. Blomberg, and C. O. Webb. 2010. Picante: R tools for integrating phylogenies and ecology. Bioinformatics **26**:1463-1464.

Lobet, G., L. Pagès, and X. Draye. 2011. A Novel Image-Analysis Toolbox Enabling Quantitative Analysis of Root System Architecture    Plant Physiology **157**:29-39.

Stegen, J. C., X. Lin, A. E. Konopka, and J. K. Fredrickson. 2012. Stochastic and deterministic assembly processes in subsurface microbial communities. The ISME Journal **6**:1653-1664.

Sugawara, M., B. Epstein, B. D. Badgley, T. Unno, L. Xu, J. Reese, P. Gyaneshwar, R. Denny, J. Mudge, A. K. Bharti, A. D. Farmer, G. D. May, J. E. Woodward, C. Médigue, D. Vallenet, A. Lajus, Z. Rouy, B. Martinez-Vaz, P. Tiffin, N. D. Young, and M. J. Sadowsky. 2013. Comparative genomics of the core and accessory genomes of 48 Sinorhizobiumstrains comprising five genospecies. Genome Biology **14**:R17.

Trachsel, S., S. M. Kaeppler, K. M. Brown, and J. P. Lynch. 2011. Shovelomics: high throughput phenotyping of maize (Zea mays L.) root architecture in the field. Plant and Soil **341**:75-87.

Wen, T., P. Xie, C. R. Penton, L. Hale, L. S. Thomashow, S. Yang, Z. Ding, Y. Su, J. Yuan, and Q. Shen. 2022. Specific metabolites drive the deterministic assembly of diseased rhizosphere microbiome through weakening microbial degradation of autotoxin. Microbiome **10**:177.

Wen, T., J. Yuan, X. He, Y. Lin, Q. Huang, and Q. Shen. 2020. Enrichment of beneficial cucumber rhizosphere microbes mediated by organic acid secretion. Horticulture Research **7**:154.

Yuan, J., J. Zhao, T. Wen, M. Zhao, R. Li, P. Goossens, Q. Huang, Y. Bai, J. M. Vivanco, G. A. Kowalchuk, R. L. Berendsen, and Q. Shen. 2018. Root exudates drive the soil-borne legacy of aboveground pathogen infection. Microbiome **6**:156.

Zeisel, A., O. Zuk, and E. Domany. 2011. FDR CONTROL WITH ADAPTIVE PROCEDURES AND FDR MONOTONICITY. The Annals of Applied Statistics **5**:943-968.
